# *De novo* sequencing and analysis of *Lophophora williamsii* transcriptome, and searching for putative genes involved in mescaline biosynthesis

**DOI:** 10.1186/s12864-015-1821-9

**Published:** 2015-09-02

**Authors:** Enrique Ibarra-Laclette, Flor Zamudio-Hernández, Claudia Anahí Pérez-Torres, Victor A. Albert, Enrique Ramírez-Chávez, Jorge Molina-Torres, Araceli Fernández-Cortes, Carlos Calderón-Vázquez, José Luis Olivares-Romero, Alfredo Herrera-Estrella, Luis Herrera-Estrella

**Affiliations:** Laboratorio Nacional de Genómica para la Biodiversidad (LANGEBIO), Centro de Investigación y Estudios Avanzados del IPN, 36500 Irapuato, Guanajuato México; Red de Estudios Moleculares Avanzados, Instituto de Ecología A.C., 91070 Xalapa, Veracruz México; Investigador Cátedra CONACyT, Instituto de Ecología A.C., 91070 Xalapa, Veracruz México; Department of Biological Sciences, University at Buffalo, Buffalo, New York 14260 USA; Departamento de Biotecnología y Bioquímica, Unidad Irapuato, Centro de Investigación y de Estudios Avanzados del IPN, 36821 Irapuato, Guanajuato México; Centro Interdisciplinario de Investigación para el Desarrollo Integral Regional (CIIDIR), Instituto Politécnico Nacional, 81000 Guasave, Sinaloa México

## Abstract

**Background:**

*Lophophora williamsii* (commonly named peyote) is a small, spineless cactus with psychoactive alkaloids, particularly mescaline. Peyote utilizes crassulacean acid metabolism (CAM), an alternative form of photosynthesis that exists in succulents such as cacti and other desert plants. Therefore, its transcriptome can be considered an important resource for future research focused on understanding how these plants make more efficient use of water in marginal environments and also for research focused on better understanding of the overall mechanisms leading to production of plant natural products and secondary metabolites.

**Results:**

In this study, two cDNA libraries were generated from *L. williamsii*. These libraries, representing buttons (tops of stems) and roots were sequenced using different sequencing platforms (GS-FLX, GS-Junior and PGM, respectively). A total of 5,541,550 raw reads were generated, which were assembled into 63,704 unigenes with an average length of 564.04 bp. A total of 25,149 unigenes (62.19 %) was annotated using public databases. 681 unigenes were found to be differentially expressed when comparing the two libraries, where 400 were preferentially expressed in buttons and 281 in roots. Some of the major alkaloids, including mescaline, were identified by GC-MS and relevant metabolic pathways were reconstructed using the Kyoto encyclopedia of genes and genomes database (KEGG). Subsequently, the expression patterns of preferentially expressed genes putatively involved in mescaline production were examined and validated by qRT-PCR.

**Conclusions:**

High throughput transcriptome sequencing (RNA-seq) analysis allowed us to efficiently identify candidate genes involved in mescaline biosynthetic pathway in *L. williamsii;* these included tyrosine/DOPA decarboxylase, hydroxylases, and *O*-methyltransferases. This study sets the theoretical foundation for bioassay design directed at confirming the participation of these genes in mescaline production.

**Electronic supplementary material:**

The online version of this article (doi:10.1186/s12864-015-1821-9) contains supplementary material, which is available to authorized users.

## Background

The “Peyote” is a small, fleshy cactus without spines that grows wild in the Mexican highlands and in the arid regions of South-western United States [1]. Peyote belongs to the genus *Lophophora,* which includes two species, *L. williamsii* and *L. diffusa* [1]. This plant is capable of producing large amounts of alkaloids with psychotropic activity, such as β-phenylethylamine (class I) or tetrahydroisoquinoline (class II), which are derived from the amino acid tyrosine [2, 3]. The function of most alkaloids in plants is unclear and their importance in metabolism is highly controversial.

Since the identification of morphine in 1806, contained in opium poppy (*Papaver somniferum*), more than ten thousand alkaloids with different properties and a variety of biological activities have been isolated from plants. Alkaloids are heterocyclic compounds that contain a nitrogen atom. The position of the nitrogen atom in the hydrocarbon ring varies among different alkaloids and different plant families. The levels of alkaloids in plants also vary from trace amounts to up to 10 % of dry weight, and a single plant species might contain over one hundred of different types. Most alkaloids are highly toxic and therefore have the potential to function in the chemical defense arsenal of plants against attack by herbivores and microorganisms. For example, nicotine (present in tobacco leaves) inhibits the growth of tobacco hornworm larvae, and the purified compound can also be applied as an effective insecticide in greenhouses. In addition, alkaloids have been suggested to serve as a storage form of nitrogen or as protectants against damage by ultraviolet light [4]. Alkaloids have traditionally been of great interest to humans because of their pronounced physiological and medical properties (e.g. morphine, atropine, quinine, caffeine and nicotine) [5–7]. Peyote mescaline (3,4,5-trimethoxyphenethylamine) is a class I hallucinogenic alkaloid, and although it is chemically unrelated to lysergic acid diethyl amide (LSD), the hallucinogenic effects of mescaline are similar to those of LSD, albeit longer lasting [8]. For centuries, North American indigenous peoples have used mescaline as a medicine, and as a part of hallucinogenic religious sacrament [9]. The ceremonial use of peyote alkaloids has masked and mythologized the potential use of peyote in modern medicine. For example, some of the illnesses treated with peyote by Mexican Natives are tuberculosis, pneumonia, scarlet fever, intestinal ills, diabetes, rheumatic pains, colds, grippe, fevers, and venereal diseases, which is why peyote is officially listed in the Mexican pharmacopoeia [10].

Although *L. williamsii* is morphologically similar to *L. diffusa*, these two species differ in stem color, the presence or absence of ribs and furrows across stems, and the color of flowers. While *L. williamsii* has blue-green or sometimes reddish-green stems with ribs and furrows and the flower is usually pink, *L. diffusa* shows yellowish-green stems without ribs and the flower is usually white. Additionally, it has been proposed that *L. williamsii* specimens can be classified into two groups according to: (i) differences in button morphology, such as size of protuberances on the epidermis of stem, (ii) levels of mescaline, and (iii) differences between their chloroplast trnL/trnF regions [11].

Next-generation sequencing technologies, such GS-FLX (also named 454) and PGM (also named Ion torrent) pyrosequencing, have the potential to dramatically increase the availability of sequence data in non-model plants that lack complete genome sequence information. In order to identify genes with potential relevance in mescaline biosynthetic pathways, we sequenced and assembled 307.2 Mpb of *L. williamsii* transcriptome data using both the GS-FLX and PGM pyrosequencing platforms. Additionally, using gas chromatography–mass spectrometry analysis (GC–MS), we provide evidence that some alkaloids present in peyote are confined to specific organs; for example, mescaline was only detected in the peyote buttons (tops of stems) but not in roots. This work provides one of the first catalogs of genes present in a medicinally relevant member of the Cactaceae family, a horticulturally important group of plants.

## Results and discussion

### Mescaline content analysis

In order to perform transcriptome analysis of mescaline-producing cacti, we collected samples in Cuatro Cienegas, Coahuila, Mexico. To confirm the identity of the collected samples, the intergenic spacer region trnL/trnF sequence was amplified using previously reported primers [11] and sequenced (ABI PRISM 3730xl). The sequence obtained was 879 bp, corresponding to the typical size reported for group 1 of *L. williamsii* species, which contain mescaline [11] (Additional file 1). To explore the content of alkaloids present in *L. williamsii*, a standard alkaloid extraction procedure (see methods) was carried out for the roots and buttons (tops of stems) from one of the peyote plants collected. The extracts were then analyzed by gas chromatography–mass spectrometry (GC-MS). Several compounds were then reliably identified by comparison against internal library spectra (Additional file 2: Figure S1 and Additional file 3: Table S1). Some of the detected compounds include hordenine, N-methylmescaline, N-acetylmescaline, pellotine, anhalonine, anhalidine, anhalonidine, O-methylanhalonidine, and lophophorine. Not all of these substances exhibit psychopharmacological activity when administered singly, but in combination, they apparently potentiate the effects of the mescaline and definitely alter some characteristics for the experience. Alkaloids such as lophophorine can be detected in extracts derived from both button and roots while some others like hordenine, which possess antibacterial properties, presumably because of their phenolic function [12], were detected only in roots (Additional file 3: Table S1). Mescaline, with retention time ≈ 8.36, was detected in buttons, but barely present in root extracts, to levels of 15.68 and 0.05 % respectively. These results show that mescaline accumulation is almost totally confined to peyote buttons.

### Sequencing and *de novo* assembly of the *L. williamsii* transcriptome

In order to explore the peyote transcriptome, we synthesized cDNA from pooled RNAs isolated from both roots and buttons. The cDNAs were used to construct sequencing libraries for two 454 Genome Sequencer platforms (GS-FLX and GS-Junior), generating a total of 492,402 sequence reads (377,522 from GS-FLX and 114,880 from GS-Junior) with an overall average read length of 230.61 and 406.31 bp, respectively (Additional file 3: Table S2). In order to identify genes with preferential expression in either roots or buttons, an additional two sequencing runs were performed using a PGM™ sequencer (Personal Genome Machine™; Life Techonologies), for which each organ (button and roots) was distinguished by using Ion barcode adapters. A total of 5,541,550 reads was generated with the PGM sequencer (2,293,355 for buttons and 3,248,195 for roots). 454-reads were masked using the SeqClean software in order to eliminate sequence regions that could cause incorrect assembly. Targets for masking included poly A/T tails, ends rich in undetermined bases, and low complexity sequences. For the Ion-reads, we first used the FASTX tool kit (http://hannonlab.cshl.edu/fastx_toolkit) to separate reads according to the specific barcodes, whereafter the barcodes were trimmed and the remaining sequences were filtered. Only high quality Ion-reads ranging from 50 to 150 bp, for which a maximum of 5 bases with a quality phred score below 17 were allowed, were used in this study. In total, 459,106 masked 454-reads and 1,979,580 high quality Ion-reads (1,147,370 from roots and 832,210 from buttons), were considered to obtain *de novo* assembly of the peyote transcriptome. An overview of the sequencing dataset is presented in Additional file 3: Table S2. The sequenced reads were assembled using MIRA [13], which is an assembler that can integrate various platform data and perform true hybrid assemblies specially for *de novo* transcriptomes [13]. This assembly resulted in a total of 129,358 transcripts (contigs) that were used in a second assembly step using CAP3 [14] to eliminate redundant sequences and increase transcript length. This assembly resulted in 63,704 contigs with lengths ranging from 0.1 to ~4.2 Kbp. Only contigs larger than 200 bp (40,436 sequences, 63.47 % of the total), for which the mean length was 564.04 bp, were considered as unigenes for further analysis (Additional file 4).

### Sequence annotation

**Fig. 1:**
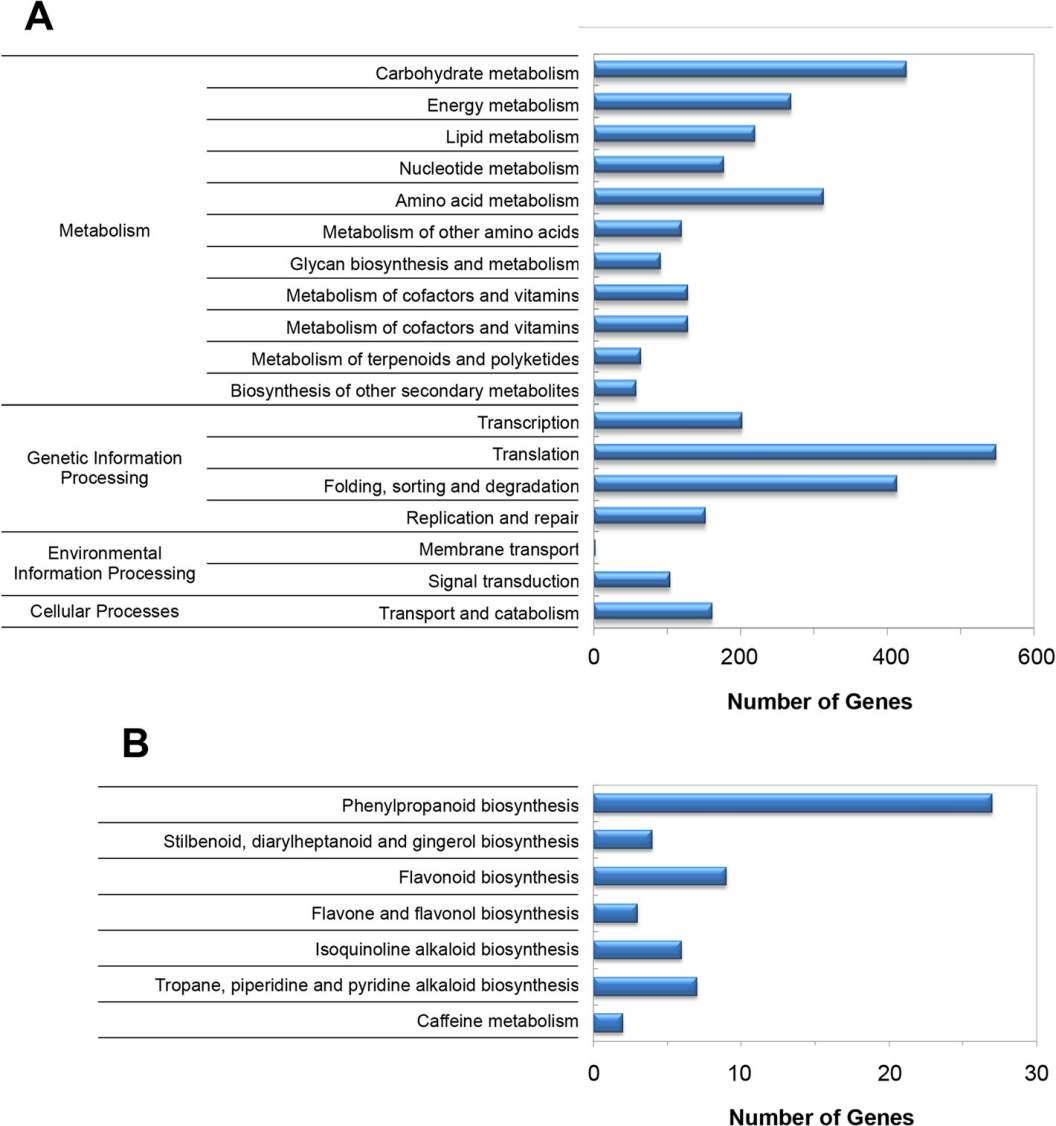
Pathway assignment based on KEGG. **a** Major categories based on molecular interaction and reaction networks. **b** Subclasses and gene distribution in the ‘Biosynthesis of other secondary metabolites’ category

The 40,436 *L. williamsii* unigenes were annotated by sequence similarity BLASTx searches against *Arabidopsis thaliana* (www.arabidopsis.org) and reference plant proteins (Refseq; comprising all green plants, ftp://ftp.ncbi.nlm.nih.gov/refseq/release/plant/) datasets. A total of 25,149 (62.19 %) unigenes showed significant similarity (e-value 10^−3^) to Arabidopsis or the RefSeq databases (Additional file 3: Table S3). The high percentage of *L. williamsii* unigenes obtained in this study that did not match the RefSeq protein database (37.81 %) indicates that there is potential for the discovery of as-yet undescribed and novel plant genes in peyote, although many of these unigenes may represent non-coding RNAs or untranslated regions (UTRs). In addition, the significance of a BLAST search depends on the length of the query sequence; therefore, short sequences are rarely matched to known genes [15], or these sequences may represent rapidly evolving parts of genes that have diverged substantially from their homologs [16]. Based on the Arabidopsis top hits, we obtained the gene ontology annotations (GOs) for 23,729 *L. williamsii* unigenes (Additional file 3: Table S4). Using the WEGO software [17] unigenes with Arabidopsis hits were assigned to gene ontology classes with 85,988 functional terms. Biological processes comprised the majority of the functional terms (39,957; 46.47 %), followed by cellular components (23,619; 27.47 %) and molecular functions (22,412; 26.06 %) (Additional file 2: Figure S2; see also Additional file 3: Table S4). In addition, using the KEGG Automatic Annotation Server (KAAS; http://www.genome.jp/tools/kaas/) from the Kyoto Encyclopedia of Genes and Genomes (KEGG) database [18], Enzyme Commission (EC) numbers [19] and KEGG Orthology (KO) codes were also associated to each annotated *L. williamsii* unigene. 5,008 unigenes having KO codes were assigned to metabolic, genetic and environmental information processing pathways (Additional file 3: Tables S3 and S5). KEGG metabolic pathways that are well-represented by *L. williamsii* unigenes belong to carbohydrate, amino acid, energy and lipid metabolism (Fig. 1a). In the subclass secondary metabolism, the greatest number of unique sequences was mapped to phenylpropanoid biosynthetic pathways, for which tyrosine is the initial precursor (Fig. 1b). This result was expected considering that *L. williamsii* classes I and II alkaloids are also derived from this amino acid.

In order to explore if the *L. williamsii* unigene database comprised a deep representation of the complex metabolic pathways that characterize plant genomes, some well-known metabolic pathways such as glycolysis/gluconeogenesis, starch and sucrose metabolism, and carbon fixation during photosynthetic process (Crassulacean acid metabolism; CAM and C4-dicarboxylic acid cycle) were reconstructed (Additional file 2: Figures S3–S5, respectively). With only a few exceptions, the proteins involved in these metabolic pathways were found in the peyote transcriptome. In most cases, more than one transcript sequence was found to encode the same enzyme. Such unique sequences may represent different fragments of a single transcript or different members of a gene family. These results indicate that the *L. williamsii* unigene database comprises a good representation of the peyote transcriptome, permitting its use as a source to discover candidate genes responsible for mescaline biosynthesis.

### Detection of preferentially expressed unigenes

Given that mescaline accumulates only in the peyote buttons, it is tempting to think that genes involved in mescaline biosynthesis are also preferentially expressed in these organs. Considering that independent samples of mRNA from *L. williamsii* isolated from button and roots were used to construct the PGM libraries, an RNA-seq approach was used to analyze the expression profile of obtained unigenes. All of 1,979,580 high quality Ion-reads (1,147,370 from roots and 832,210 from buttons) were mapped independently to the *L. williamsii* transcriptome, and an expression profile matrix containing the unigenes (rows) and the number of mapped reads in each normalized organ-specific transcriptome (columns), was created (Additional file 3: Table S6).

It has been argued that a high number of reads (in the tens of millions) are required to perform gene expression analysis [20, 21]. Considering the number of sequences generated in the present study, we used relative frequency values (counts divided by the effective library sizes) to make reads counts comparable among samples. This method has been previously used for the efficient detection of differentially expressed genes [22, 23] (see methods for more details). The significance of differential gene expression between buttons and roots was determined using the likelihood ratios (R) method described by Stekel et al. [24]. This approach is based on a single statistical test that can be used to describe the extent to which a gene is differentially expressed between libraries. Briefly, all unigenes (and their corresponding read-counts values) were used to calculate a log likelihood ratio that trends asymptotically to a χ2 distribution in which R values ≥ 12 can be considered significantly preferentially expressed genes. Additionally, a fold change of at least 2-fold (buttons/roots) was also considered. A total of 652 unigenes was identified as preferentially expressed, 400 in buttons and 281 in roots (Additional file 3: Table S7). Interestingly, theorgan-enriched, differentially expressed genes reflect known organ-specific biological activities. Sixty unigenes (15 % of the total) selected as preferentially expressed in buttons are involved in energy metabolism (photosynthesis, oxidative phosphorylation and carbon fixation). Considering these results we suggest that the gene expression profiles found for each library can be used for the identification of genes involved in specific metabolic pathways.

### Crassulacean acid metabolism

Crassulacean acid metabolism, which often operates in species subjected to low-water conditions, is a key adaptation in flowering plants. This metabolism increases the efficiency of photosynthesis by increasing CO_2_ and carbon capture in plant tissues, where temporal separation of the photosynthetic stages prevents water loss due to transpiration. This mechanism is mediated mainly by phosphoenolpyruvate carboxylase (PEPC, which catalyzes nocturnal CO_2_ fixation and producing oxaloacetate), phosphoenolpyruvate carboxylase kinase (PEPK, which controlsphosphorylation state of PEPC), malate dehydrogenase (MDH, which reduces oxaloacetate to malate), NADP-dependent malate dehydrogenase (NADP-ME, which decarboxylates malate in the cytoplasm), and pyruvate phosphate dikinase (PPDK, which converts pyruvate into PEP) [25, 26]. At the end, CO_2_ is available as a substrate for RUBISCO in the Calvin cycle. Besides these enzymes, a set of sugar transporters, Na^+^/H^+^ antiporters and aquaporins are needed to maintain favorable conditions for CAM [27].

Available genetic and genomic resources in CAM plants, including *Ananas comosus* L, *Agave deserti*, *Agave tequilana*, *Opuntia ficus-indica,*and *Mesembryanthemum crystallinum,* have revealed CAM genetic determinants [27–30], some of which have been proposed to encode homologs of candidate regulatory proteins such as TOC1, CCA1, RPP9, and ZTL [30–32]. TOC1 binds to the G-box and EE-motif promoter regions of genes involved in both the morning and evening transcription-translation feedback loops that drive the plant circadian clock; these genes include *PRR9* and *CCA1* in the morning feedback loop. Discrete induction of *TOC1* gene expression results in reduced *CCA1* and *PRR9* expression, indicating that TOC1 plays a repressive rather than stimulatory role in regulating circadian gene expression [33]. The dark-dependent degradation of TOC1 protein requires expression of *ZTL*, and is prevented by inhibiting the proteosome pathway; therefore, the TOC1-ZTL interaction is important in the control of *TOC1* and is responsible for the regulation of circadian period [34]. Furthermore, recent work in Orchidaceae demonstrated that CAM has evolved at least 10 times independently in different families including both monocots and eudicots [35]. A full understanding of CAM biology requires the identification and analysis of the genes that code for enzymes and regulators of this mechanism. Sequencing efforts in the present work have captured most of the putative orthologous genes of CAM metabolism in *L. williamsii*. Additional file 2: Figure S3 shows the enzymes involved in CAM whose genes are identified in our unigene dataset, including phosphoenolpyruvate carboxylase [EC:4.1.1.31], pyruvate, orthophosphate dikinase [EC:2.7.9.1], malate dehydrogenase (oxaloacetate-decarboxylating) (NADP^+^) [EC:1.1.1.40], malate dehydrogenase [EC:1.1.1.39]. Also, several sequences in the unigene dataset were annotated as sugar transporters, antiporters or aquaporins (Additional file 3: Table S5).

Among the annotated unigenes, *UN03078* and *UN24453* were highly similar to Arabidopsis *ZTL* (AT5G57360) and *CCA1*, respectively (Additional file 3: Table S3). These unigenes are unique sequences matching known circadian clock regulators related with CAM. It remains to be determined whether these sequences indeed represent the orthologs of Arabidopsis *ZTL* and *CCA1*, and whether they play a role in the circadian regulation of CAM in peyote.

### Identification of *Lophophora williamsii* unigenes with potential relevance to the biosynthetic pathway of mescaline

**Fig. 2:**
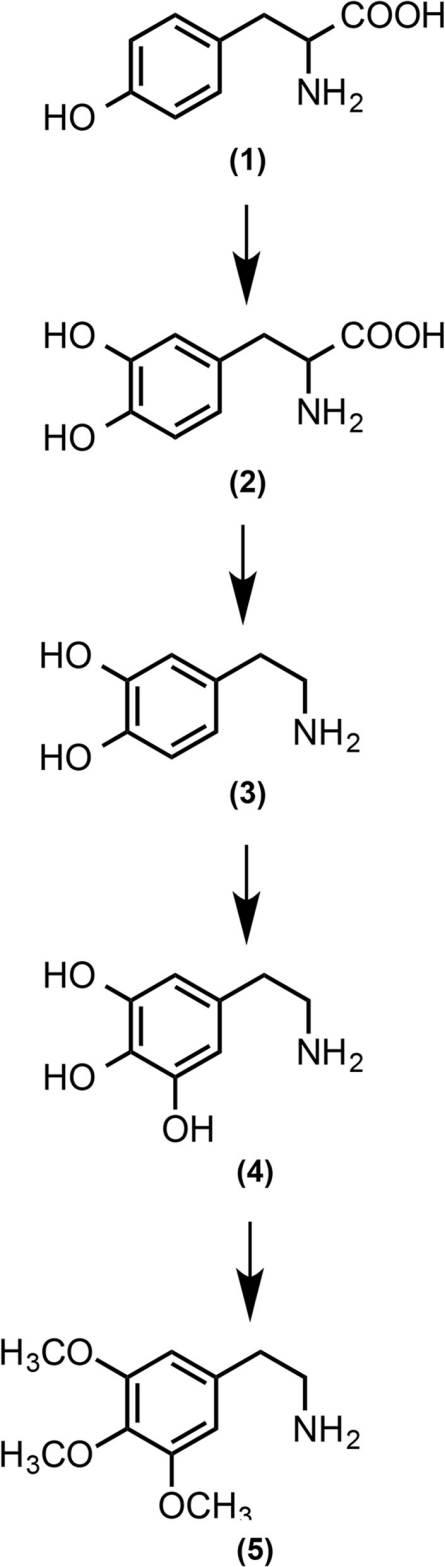
Biosynthetic pathway of mescaline in *Lophophora williamsii*. The main intermediates in the pathway are tyrosine (1), dopa (2) dopamine (3) 3,4,5-trihydroxy-β-phenyethylamine (4), and mescaline (5). This figure was modified from [84]

The *in vivo* pathway for the synthesis of mescaline was first proposed by Paul *et al.*, [36] (Fig. 2). Starting from tyrosine (1), the main intermediates are dopa (2), dopamine (3), and 3,4,5-trihydroxy-β-phenyethylamine (4), resulting in mescaline (5). These reactions include hydroxylation of (1) to (2), decarboxylation to (3), hydroxylation to (4) and methylation to (5). All enzymes required for the formation of tyrosine from glucose are represented in the *L. williamsii* unigene database (Additional file 2: Figure S6). Enzymes selected as candidates for involvement in mescaline biosynthesis were postulated based on the high sequence similarity of homologous genes and their expression profiles.

### Tyrosine/DOPA decarboxylation

**Fig. 3:**
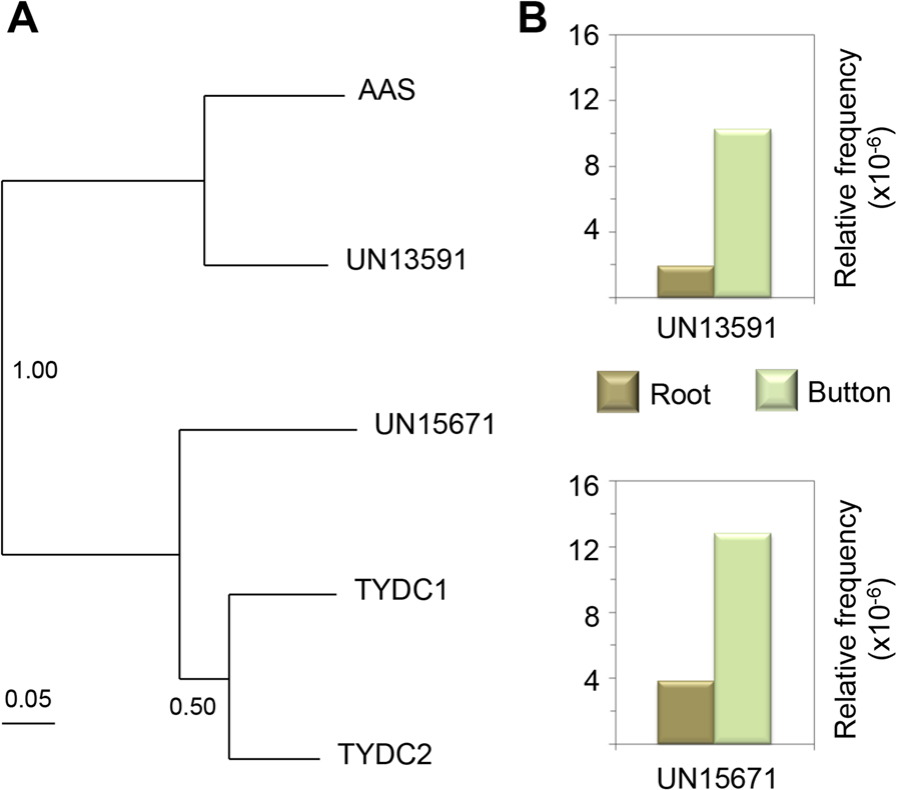
**a** Maximum Likelihood (ML) phylogenetic tree based on amino acid sequences of the conserved pyridoxal-dependent decarboxylase domain. The alignment includes the deduced protein sequences of the *UN13591* and *UN15671 * unigenes of *L. williamsii*, *A. thaliana* aromatic aldehyde synthase (ATAAS), and *P. somniferum* tyrosine/DOPA decarboxylases (TYDC1 and TYDC2). Branch numbers represent the robustness of the three analyzed by approximate likelihood-ratio test (aLRT). **b** Expression patterns of *L. williamsii* unigenes in buttons and roots derived from RNA-seq analysis. RNA-seq data are shown as relative frequency values

Opium poppy (*Papaver somniferum*) is the source of several pharmaceutical benzylisoquinoline alkaloids including morphine, codeine and sanguinarine. The biosynthesis of these alkaloids starts with the condensation of two tyrosine derivatives, dopamine and 4-hydroxyphenylacetaldehyde. The formation of dopamine involves the decarboxylation of tyrosine [EC:4.1.1.25] and/or dihydrophenylalanine (DOPA) by tyrosine/DOPA decarboxylase [EC:4.1.1.28] [37]. The members of the tyrosine/dopa decarboxylase (TYDC) gene family in opium poppy can be categorized into two subgroups according to their sequence homology. Representative members of each subgroup (*TYDC1* and *TYDC2*) share 73 % amino acid identity, and both encoded enzymes exhibit L-dopa and L-tyrosine decarboxylase activities [37]. Three different *L. williamsii* unigenes (UN08840, UN13591 and UN15671) were annotated as homologous to Arabidopsis aromatic aldehyde synthase (ATAAS; AT2G20340). This enzyme catalyzes the conversion of phenylalanine and 3,4-dihydroxy-L-phenylalanine to phenylacetaldehyde and dopaldehyde, respectively [38]. According to the best BLAST hits annotation derived from the RefSeq-plant database, these unigenes are homologs of tyrosine decarboxylase (*Medicago truncatula*), a predicted protein in *Populus trichocarpa,*and a tyrosine/DOPA decarboxylase in *Glycine max*. Part of the distinctive pyridoxal-dependent decarboxylase conserved domain (PF00282) was identified in both UN13591 and UN15671 peyote unigenes by motif/domain search against the Pfam database (http://pfam.janelia.org). In contrast, the coding region represented in unigene *UN08840* is homologous only to the carboxy-terminal portion of the ATAAS protein. Using the SeaView program [39], the protein-coding nucleotide sequences were aligned based on their corresponding amino acid translations (Additional file 2: Figure S7 and Additional file 5). A phylogenetic tree of peyote unigenes based on their pyridoxal-dependent decarboxylase domain sequence, including the TDYC1, TDYC2 and ATAAS proteins, was generated (Fig. 3). UN15671 was grouped with opium poppy *TDYC* sequences while the *UN13591* unigene was grouped in the same clade with *ATAAS*. These data suggest that at least an ortholog to the *P.somniferumTYCD* (represented by the unigene UN15671) is present in *L. williamsii*, which could be responsible for tyrosine conversion to dopamine in this species. Interestingly, according to the relative frequency values obtained, these peyote unigenes showed higher levels of transcripts in buttons than in roots (Fig. 3).

### Hydroxylation of aromatic compounds

Considering that during mescaline biosynthesis, dopamine is hydroxylated to form 3,4,5-trihydroxy-β-phenyethylamine, the *L. williamsii* transcriptome was surveyed in order to identify homologs of some biochemically characterized plant enzymes capable of introducing the hydroxyl group (-OH) into aromatic compounds (reviewed in [40]), especially those whose substrate is an aromatic amino acid that contains a single benzene ring.

Polyphenol oxidases (PPO) catalyze the *O*-hydroxylation of monophenols (phenol molecules in which the benzene ring contains a single hydroxyl substituent) to *O*-diphenols (phenol molecules containing two hydroxyl substituents). They can also further catalyze the oxidation of *O*-diphenols to produce *O*-quinones [41]. Tyrosinases are bifunctional PPOs that catalyze the *O*-hydroxylation of monophenols and subsequent oxidation of *O*-diphenols to quinines [42]. Thus, tyrosinases accept both mono- and di-phenols as substrates. The hydroxylation ability of the enzyme is also referred to as cresolase or monophenolase activity [EC:1.14.18.1], and the oxidation ability as catecholase or diphenolase activity [EC:1.10.3.1]. The monophenolase activity of tyrosinases is known to be the initial rate-determining reaction [42, 43]. In tyrosinase-catalyzed reactions, molecular oxygen is used as an electron acceptor that is reduced to water. Tyrosinases and their corresponding genes have been characterized from various sources, including bacteria, fungi, plants and mammals [41, 44]. A sequence comparison of recently published tyrosinases reveals high heterogeneity concerning their length and overall identity. However, highly conserved regions among all tyrosinases can be found in the active site domain [44, 45]. The peyote unigene *UN14261* was annotated as a homolog of *Glycine max* PPO (RefSeq accession number XP_003522849.1). A Motif/domain search revealed that a KFDV-containg PPO1 C-terminal domain (PF122143) can be identified in the translated sequence corresponding to this peyote unigene (Additional file 2: Figure S8 and Additional file 6). This domain family is found in association with the common central domain of tyrosinase (PF00264), and is typically between 138 and 152 amino acids in length. Even though the functional significance of these domains is not known, many members of this family are plant or plastid polyphenol oxidases with the highly conserved sequence motif KFDV, from which the name derives [46].

An in-depth blast search, for which the coding sequence of the *POP1* KFDV domain was used as reference (e-value 10^−03^), revealed that no additional PPOs could be identified in the peyote unigene dataset. Although unigene *UN14261* could be considered as an interesting candidate to participate in mescaline biosynthesis, in contrast to unigene *UN15671* (an ortholog of *P.somniferum* TYDC proteins), its transcript levels indicate that *UN14261* is preferentially expressed in roots rather than buttons (Additional file 3: Table S6).

Folate- or tetrahydropterin-dependent aromatic amino acid hydroxylases (AAHs) are an additional group of enzymes localized in chloroplasts capable of catalizing the *O*-hydroxylation of the benzene ring of aromatic amino acids [47–49]. However, homologs of this family, which comprise phenylalanine [EC:1.14.16.1], tyrosine [EC:1.14.16.2], and tryptophan [EC:1.14.16.4] hydroxylases [48, 49], are not present in our *L. williamsii* unigene dataset. This was expected considering that thus far, such genes have been identified only in non-flowering plants [49].

α-keto acid dependent enzymes catalyze dealkylations, epimerizations and halogenations, hydroxylation, and a variety of oxidations [50]. Fe^2+^/α-keto acid-dependent oxygenases represent a group of dioxygenases [EC:1.14.11.–] that use α-ketoglutarate (α-KG) as substrate, incorporating two oxygen atoms from O_2_ into two different substrates where one atom is transferred to the actual substrate, and the second one to the α-KG acting as the co-substrate (reviewed in [50]). A large variety of compounds such as flavonoids and alkaloids may be synthesized by Fe^2+^/α-KG-dependent dioxygenases in plants [51–53]. Other types of enzymes such as 4-hydroxyphenylpyruvate dioxygenase (HPPD; EC:1.13.11.27) have no relationship in sequence but do show a similar reaction mechanism to Fe^2+^/α-KG hydroxylases, and these enzymes also contain ferrous iron (Fe^2+^) in the active site, but the α-keto acid that is decaoxylated is part of the substrate and the hydroxylation is associated with an ‘NIH’ shift (a chemical rearrangement wherein a hydrogen atom on an aromatic ring undergoes an intramolecular migration primarily during a hydroxylation reaction; [54, 55]). 4-Hydroxyphenylpyruvate dioxygenase, which catalyzes the formation of 2,5-dihydroxyphenylacetate (homogentisate), has been found in all aerobic forms of life where it is involved in tyrosine metabolism [54, 56].

**Fig. 4:**
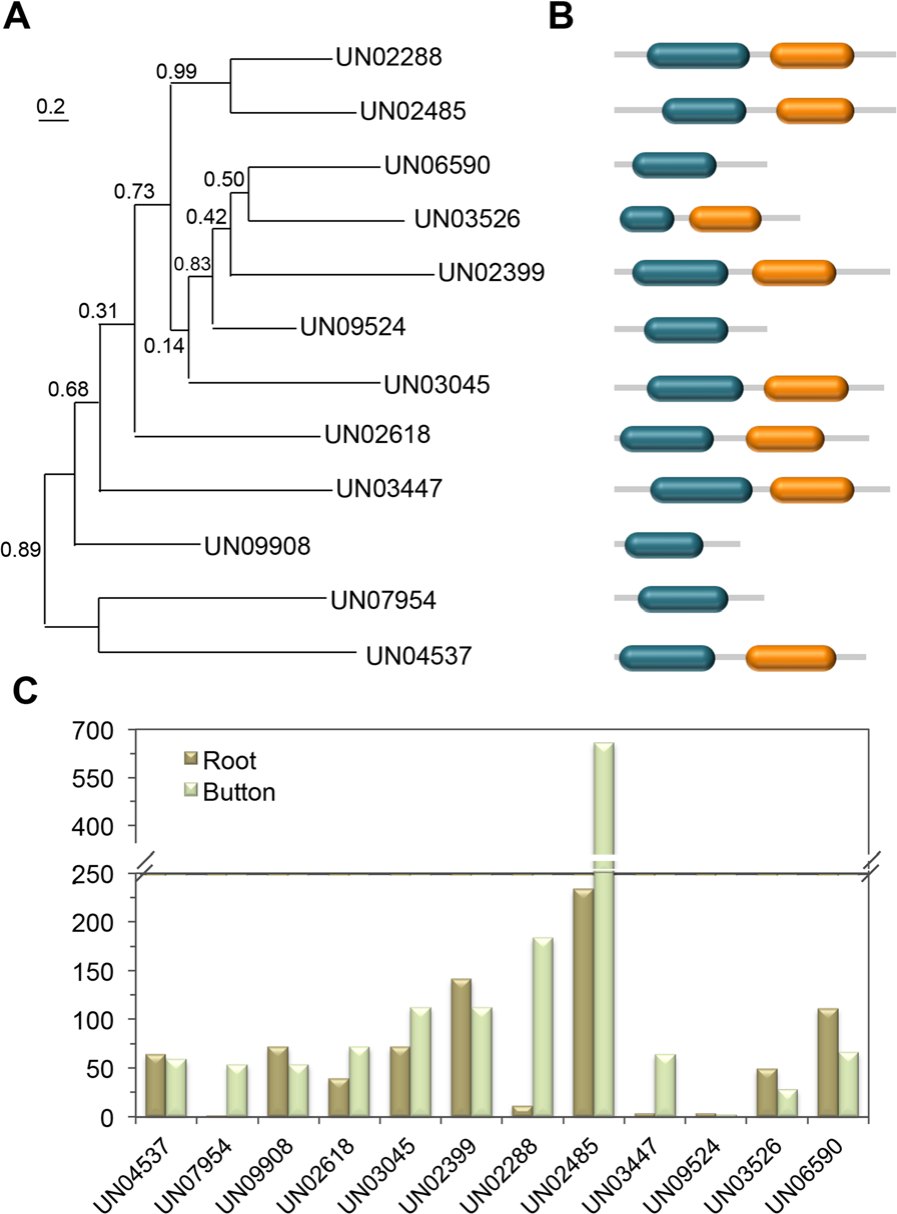
Phylogenetic relationships, primary protein structures and expression patterns of *L. williamsii* Fe2+/α-keto acid-dependent oxygenases. **a** ML un-rooted phylogenetic tree. **b** Schematic representation of domain structure along the length of protein. DIOX-N (PF14226) and 2OG-FeII-Oxy (PF03171) domains are represented by green and orange boxes, respectively. **c** Expression analysis of *L. williamsii* unigenes in buttons and roots performed by RNAseq

Through a BLAST search based on the distinctive 2OG-FeII-Oxy domain (PF03171) of the Fe^2+^/α-KG oxygenases, a total of 16 peyote unigenes were identified as members of this family. These unigenes were aligned against their plant homologs, identified in the annotation process as the top BLAST hits against the RefSeq database. To identify the coding sequences in their correct open reading frames, we used the known plant homologs as references and protein-coding nucleotide sequences were aligned based on their corresponding amino acid translations using the SeaView program (Additional file 7). Among 12 peyote unigenes, complete (or almost complete) coding sequences (CDS) ranging between 510 and 1,083 bp were identified. Additionally, a highly conserved non-heme dioxygenase N-terminal domanin (DIOX-N; PF14226), commonly associated with 2-oxoglutarate/Fe(II)-dependent dioxygenase proteins, was identified in the peyote unigene set (7 in total). The phylogenetic relationships among peyote Fe^2+^/α-KG oxygenases, and their expression profiles, were analyzed (Fig. 4). Six of these unigenes showed highest expression levels in buttons, and four of them had two-fold or greater transcription in roots (*UN07954*, *UN02288, UN02485* and *UN03447*).

With regard to HPPD proteins [EC:1.13.11.27], a similar approach was used to identify putative homologs. Using the glyoxalase domain (PF00903), a single putative *L. williamsii* HPPD CDS was identified (UN03443; Additional file 8). The predicted protein showed around 77.0 % amino acid identity compared with the published *Medicago truncatula* HPPD. The relative abundance values indicate that the transcripts of this HPPD are almost 200-fold higher in roots than in the peyote buttons (Additional file 3: Table S6).

Considering the results described above, it is proposed that one of the Fe^2+^/α-KG oxygenases expressed preferentially in buttons is a prime candidate for involvement in the hydroxylation of dopamine during mescaline biosynthesis.

### *O-*methyltransferases of *L. williamsii*

**Fig. 5:**
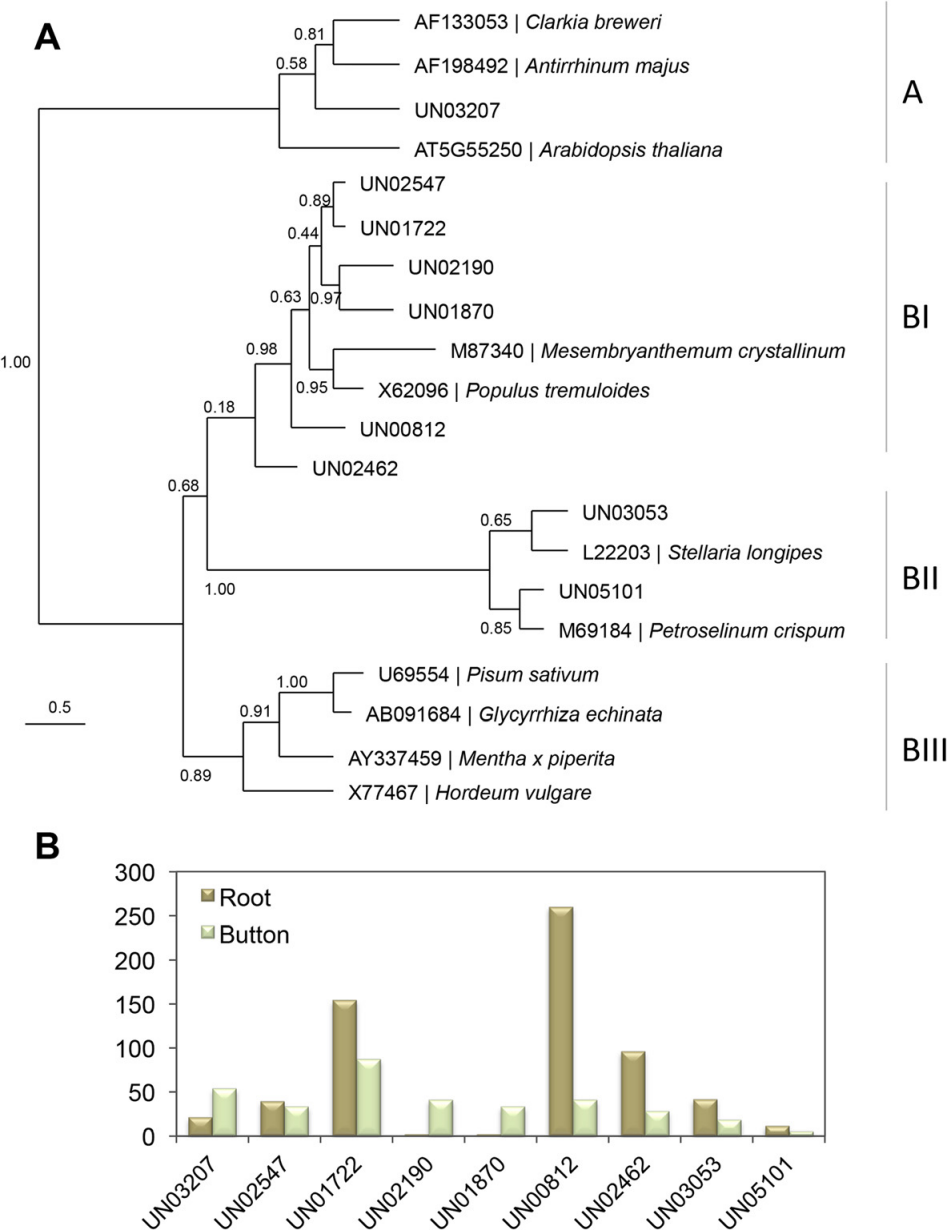
Expression patterns of OMT peyote unigenes and the phylogenetic relationships of their deduced proteins with those from various other plant species. **a** Phylogenetic relationship of plant OMTs. **b** Expression profile of OMTs genes in buttons and roots of *L. williamsii* plants

Enzymatic *O*-methylation consists of the transfer of the methyl group of a common co-substrate such as *S*-adenosyl-*L*-methionine (AdoMet) to the hydroxyl group of an acceptor molecule. This process is catalyzed by an *O-*methyltransferase (OMT) [EC 2.1.1.6.x], a group of enzymes that methylate a wide range of compounds with a high degree of selectivity [57, 58]. These enzymes are present in diverse organisms, including bacteria, fungi, plants and mammals. However, a few OMTs have been shown to be multifunctional enzymes that catalyze the methylation of structurally related compounds such as phenylpropanoids and flavonoids [59–63]. In plants, *O-*methyltransferases constitute a large family of enzymes. Novel OMT-like gene sequences have been reported using a framework phylogenetic tree encompassing 61 biochemically characterized plant OMT protein sequences for improved prediction of their putative function [64]. In order to identify OMT-like sequences, these proteins were used as reference in a BLAST similarity search (e-value 10^−06^) against the *L. williamsii* unigene dataset. A total of eleven OMT-like sequences (unigenes *UN00812*, *UN01722*, *UN01870*, *UN02190*, *UN02462*, *UN02547*, *UN03053*, *UN03207*, *UN05101*, *UN06792* and *UN28302*), in which the CDS for the *O-*methyltransferase conserved domain (PF00891) was present, were found in the peyote transcriptome database. To identify the coding sequences in their correct open reading frames, these peyote unigenes were aligned based on their corresponding amino acid translations against their best-blast-hits homologs (Additional file 9). With only two exceptions (*UN28302* and *UN06792*), these unigenes contain complete (or almost complete) coding sequences. A phylogenetic tree was generated in which the proteins derived from these peyote CDS and their homologs were included (Fig. 5).

The phylogenetic structure of the *L. williamsii* OMT gene family was similar to that previously reported by Lam *et al.* [64]. Two distinct monophyletic lineages were recognized (A and B, respectively), one of which (clade B), includes three sister clades (BI-BIII). With only one exception (clade BIII), the remaining clades contain at least one putative OMT identified in the peyote transcriptome (Fig. 5). Clade A contains plant OMTs with *S*-adenosyl-*L*-methionine (SAM) dependent methyltransferase activity, which acts on a diverse group of metabolites including salicylic acid, benzoic acid and indole-3-acetic acid (IAA) (identified in the species *Clarkia breweri, Antirrhinum majus* and *Arabidopsis thaliana*, respectively) [65–67].

Clade BI contains six different peyote unigenes (*UN00812, UN01722, UN01870, UN02190, UN02462* and *UN02547*) grouped with only two different plant OMTs: one lignin bispecific caffeic acid/5-hydroxy ferulic acid OMT (from *Populus tremuloides*) [68] and one flavonoids/caffeoyl-CoA OMT (from *Mesembryanthemum crystallinum*) [63]. Clade BII contains two peyote unigenes (*UN03053* and *UN05101*) that were grouped with two plant caffeoyl-CoA 3-o-methyltransferases (from *Stellaria longipes* and *Petroselinum crispum*; respectively) [69, 70]. Finally, clade BIII, which contained no peyote unigenes, includes some flavonoid OMTs [58, 71–73].

Mescaline biosynthesis in peyote plants requires the triple-methylation of 3,4,5-trihydroxy-β-phenyethylamine (in Fig. 2 exemplified by reaction of (4) to (5)). Considering the chemical structure of substrates such caffeoyl-CoA, 5-hydroxy ferulic and/or caffeic acid, and that plant OMTs may have specific or promiscuous activities, we propose that 3,4,5-trihydroxy-β-phenyethylamine could serve as the methyl acceptor (substrate) for one or more *L. williamsii* OMTs classified in the BI and/or BII monophyletic lineages, especially those whose transcript levels are more abundant in buttons than in peyote roots (Fig. 5). Interestingly, only three peyote OMTs showed increased levels of transcripts in buttons compared to roots.

*UN03207* was resolved as a member of clade A (SAM dependent methyltransferases), and even though the abundance of the transcript was calculated to be 2.6-fold higher in buttons than in roots, transcripts related to this OMT were detected in both organs. This was expected since metabolites such as salicylic acid, benzoic acid, and jasmonic acid play crucial roles in numerous pathways, controlling the way in which plants grow and develop [74]. With respect to unigenes *UN02190* and *UN01870*, which were grouped in clade BI as two closely related OMTs, their transcript levels were quite low in roots and 20-fold higher in buttons. Based on these results, we propose that *UN02190, UN01870*, or both unigenes could be considered as primary candidates to belong to the mescaline biosynthesis pathway.

### Real-time quantitative PCR verification

Transcriptional regulation revealed by RNA-seq data was confirmed by real-time quantitative reverse transcription PCR (qRT-PCT). A total of seven peyote unigenes was selected as primary candidate genes involved in mescaline biosynthesis (one TYDC, four Fe^2+^/α-KG oxygenases and two OMTs, respectively). A linear regression analysis showed an overall correlation coefficient of R = 0.796, which indicates a good correlation between transcript abundance assayed by real-time PCR and transcription profiles revealed by RNA-seq data (Additional file 2: Figure S9).

## Conclusions

The *L. williamsii* transcriptome provides a detailed genomic window on metabolic processes that are carried out in a cactus plant capable of accumulating high levels of alkaloids. The transcriptome of buttons (which are photosynthetic organs) and roots of peyote vary significantly in terms of the genes that can be identified in each and their consequent expression profiles. These variations could be responsible, at least in part, for the identities of the organs but also could explain differences in terms of the metabolites that are accumulated. Most genes involved in CAM metabolism, typical of slow-growing cacti in desert regions, were identified and can be used as starting points to study the regulation of genes that allow CO_2_ fixation and stomatal aperture during nighttime, as opposed to C3 and C4 plants that carry out this process during the day. Genes putatively involved in the biogenesis of mescaline were identified in this work by comparing their expression levels in button and roots. This report provides a catalogue of specific candidate genes that can be further expressed in heterologous systems in order to confirm their putative roles in mescaline biosynthesis.

## Methods

### Plant material

Since *L. williamsii* is listed on CITES (the Convention on International Trade in Endangered Species of Wild Fauna and Flora; http://www.cites.org) Appendix II and it is legally protected in Mexico by the national list of species at risk of extinction, NOM-059-SEMARNAT-2010, where it is listed under the category “subject to special protection” (Pr; SEMARNAT 2010), the plants used in this study were collected in the Coahuila desert (Cuatrociénegas municipality) according to permissions granted by Mexican authorities (DE/5336/2007 official letter issued by Subsecretaría de gestión para la protección ambiental, Dirección general de protección contra riesgos sanitarios, and Dirección general de la vida silvestre).

### Alkaloid extraction

15 g of both powdered samples (roots and buttons, respectively) were extracted at room temperature three times with EtOH (300 ml) for 48 h each time with stirring. The combined ethanol extracts were filtered and evaporated *in vacuo* to give 1915.4 and 1779.56 mg of oily residues, respectively. The residues were dissolved in H_2_O, made alkaline with concentrated ammonia (pH 9) and extracted twice with CHCl_3_ and once with CHCl_3_:EtOH (3:1). The combined extracts were dried over anhydrous Na_2_SO_4_, filtered and evaporated to dryness to yield 463.9 mg from roots and 431.0 mg from buttons, respectively, of total alkaloids.

### Gas chromatography–mass spectrometry

A gas chromatograph (Hewlett Packard 6890) coupled to a quadrupole electron impact ionization mass spectrometer (Hewlett Packard 5973) was used. The obtained mass spectra were compared with the NIST v 2008 Mass Spectra Data Base (National Institute of Standards and Technology, U.S. Department of Commerce). The GC oven temperature program was as follows: initial temperature 150 °C, increasing at a rate of 4 °C/min. to 280 °C and maintained for 20 min. Injector temperature was set to 250 °C. At the MS the ionization source was set at 250 °C and the quadrupole at 130 °C. A 2 μl aliquot of the extract was injected in split-less mode injection into a HP-5 MS capillary column (30 m × 0.25 mm × 0.25 μm Agilent Technologies, CA, USA) using Helium, chromatographic grade as carrier gas at 1 mL/min.

### RNA extraction, library construction, pyrosequencing and *de novo* assembly

Total RNA was isolated from roots and buttons using the Trizol reagent (Invitrogen) and re-purified with the RNeasy kit (Qiagen) following the manufacturer’s recommendations. RNA purity was checked using the Agilent 2100 Bioanalyzer RNA 6000 Nano Assay chip (Agilent Technologies, Stockport, U.K). cDNA synthesis was performed from 3.5 μg of total RNA using the Message Amp-II kit (Ambion, Foster City, CA) following the manufacturer’s protocol as described earlier [75]. cDNA derived from buttons and peyote roots was pooled (using the same amount of each) and sequencing on the GS-FLX and GS-Junior platforms (one run per equipment). Non-pooled cDNA was used to prepare independent libraries from buttons and roots (represented by Ion barcode adapters). cDNA samples were prepared with the Ion Xpress™ Template Kit (Life Technologies) according to the Ion Xpress™ Template Kit User Guide and sequenced on a Personal Genome Machine™ (PGM™) sequencer using two 3.18 semiconductor chips.

After a filtering process, high quality reads generated with both GS (454) and PGM (IonTorrent) technologies were pooled and subjected to *de novo* assembly using the MIRA assembler [13] and the resultant contigs were re-assembled with CAP3 [14]. As a result, 40,436 non-redundant contigs (unigenes) ranging from 200 to 4,170 bp in length were obtained. Files containing sequence reads and quality scores were deposited in the Short Read Archive of the National Center for Biotechnology Information (NCBI) [BioProject PRJNA261064].

### Annotation of the *Lophophora williamsii* unigenes

The unigenes were compared against the *Arabidopsis thaliana* (http://www.arabidopisis.org/) and RefSeq (ftp://ftp.ncbi.nlm.nih.gov/refseq/release/plant/) protein databases. In both alignments, a cut-off e-value of 10^−3^ and bit score ≤ 30 was applied and only the top blast hit was considered. Top protein matches from Arabidopsis or additional plant proteins were assigned to each of the *L. williamsii* unigenes. The gene ontology (GO) functional classes and pathways for each unigene were assigned based on Arabidopsis GO SLIM and pathway annotation (ftp://ftp.arabidopsis.org/home/tair/Ontologies/). Additionally, the unigenes were also analyzed using the KEGG Automatic Annotation Server (KAAS [76]; http://www.genome.jp/tools/kaas/). The bi-directional best hit (BBH) method was used to provide annotations of KEGG Orthology (KO) codes. Enzyme Commission (EC) numbers were also assigned based on the annotations extracted from Kyoto Encyclopedia of Genes and Genomes (KEGG) [77]. Finally, conserved domains in the selected unigenes were confirmed by motif/domain search against the Pfam database (http://pfam.janelia.org).

### Comparative analysis of transcript levels

The Ion-reads were aligned using Bowtie [78] to the *L. williamsii* assembled transcriptome. More than 95 % of total reads from each peyote organ (button and root) were mapped back to the peyote unigenes, nearly to 65 % with a single match. Read hits per unigene (reads counts) were normalized using the relative frequency of reads, i.e., the number of reads mapped for a given unigene relative to the total number of reads in a specific library. In order to identify unigenes with significant differences in their transcripts levels, the R statistical differential expression value was calculated as described by Stekel et al. [24].

### Phylogenetic analysis

Phylogenetic analyses were performed in a maximum likelihood (ML) framework using SeaView v2.4 software [39], which drives the Muscle [79] (for alignment) and PhyML [80] (for phylogenetic analysis) programs. The PhyML option was used under LG (Le and Gascuel) model [81]. Equilibrium frequencies, topologies, and branch lengths were optimized, the starting tree was determined using BioNJ, and both nearest-neighbor interchange (NNI) and subtree pruning and regrafting (SPR) algorithms for tree searching were used. Branch robustness was analyzed by approximate likelihood-ratio test (aLRT) [82].

### Evaluation of genes expression by qRT-PCR

A total of 1 μg of RNA was reverse transcribed for first-strand cDNA synthesis using SuperScript III reverse transcriptase (Invitrogen) according to the manufacturer’s instructions. Gene-specific primer pairs (Additional file 3: Table S8), which were designed using the Primer3 v.0.4.0 web tool (http://bioinfo.ut.ee/primer3-0.4.0/primer3/), were used for real-time PCR. Reactions were performed with the SYBR Green PCR Master Mix (Applied Biosystems) in an ABI PRISM 7500 Fast Real-time system. Actin was used as the standard to normalize the content of cDNA and the 2^-ΔΔCt^ method [83] was employed to compare button and root samples. Ten microliters of the reaction mixture was added to each well. The thermal cycling program was set to 50 °C for 2 min, 95 °C for 1 min, and 40 cycles of 95 °C for 15 s and 60 °C for 1 min. The real-time PCR was conducted with at least three technical replicates for each sample, and data are indicated as means ± standard error (SE).

## Additional files

Additional file 1: TrnL-trnF intergenic spacer. (FASTA 978 bytes) Additional file 2: Figure S1. GC-MS chromatograms obtained from ethanol extracts of buttons **(A)** and roots **(B)** of *L. williamsii.* **Figure S2.** Gene ontology classification of the *L. williamsii* transcriptome. **Figure S3.** The carbon fixation pathway in photosynthetic organisms. **Figure S4.** Glycolysis / Gluconeogenesis biosynthesis pathway reconstructed based on the *de novo* assembly and annotation of the *L. williamsii* transcriptome. **Figure S5.** Starch and sucrose metabolic pathway in *L.williamsii*. **Figure S7.** Alignment of pyridoxal-dependent decarboxylase conserved domains in the deduced amino acid sequences of the *UN13591* and *UN15671 L. williamsii* unigenes. **Figure S8. (A)** Alignment of the PPO1-KFDV C-terminal conserved domain of the *UN14261 L. williamsii* unigene and *Glycine max* PPO1 (XP_003522849.1). **(B)** Expression analysis of the *L. williamsii* unigene by RNA-seq. **Figure S9.** Real-time PCR validation of RNA-seq results. (PPTX 877 kb) Additional file 3: Table S1. Alkaloids found in the ethanol extracts of buttons and roots of *L. williamsii*. **Table S2.** Reads statistics of the *L. williamsii* dataset. **Table S3.** Annotation of *Lophophora williamsii* unigenes. (TAIRv10; http://www.arabidopsis.org/ and NCBI; ftp://ftp.ncbi.nih.gov/refseq/release/plant/ **Table S4.** Funtional classification of *L williamsii* unigenes based on Arabidopsis GO annotation. **Table S5.** Detailed information for *L. williamsii* unigenes mapped on KEGG pathways. **Table S6.** Expression profile matrix of *L. williamsii* unigenes. **Table S7.** Preferentially expressed unigenes in specific organs (buttons and roots) of *L. williamsii*. (XLSX 16694 kb) Additional file 4:
***L. williamsii*** **unigenes.** (ZIP 7124 kb) Additional file 5: Alignment of Pyridoxal DeC protein domain. (FASTA 2 kb) Additional file 6: Alignment of PPO1-KFDV protein domain. (FASTA 1009 bytes) Additional file 7: Alignment of αKG-dependent dioxigenase proteins. (FASTA 33 kb) Additional file 8: Alignment of HPP dioxygenase proteins. (FASTA 2 kb) Additional file 9:
**Alignment of** ***O*** **-methyltransferase proteins.** (FASTA 25 kb)
