# Supplementary material for: De novo sequencing and analysis of Lophophora williamsii transcriptome, and searching for putative genes involved in mescaline biosynthesis
Source: BMC Genomics. 2015 Sep 2;16(1):657. doi: 10.1186/s12864-015-1821-9 (PMC4557841; doi:10.1186/s12864-015-1821-9)
Supplement: Additional file 2: Figure S1. — GC-MS chromatograms obtained from ethanol extracts of buttons (A) and roots (B) of L. williamsii. Figure S2. Gene ontology classification of the L. williamsii transcriptome. Figure S3. The carbon fixation pathway in photosynthetic organisms. Figure S4. Glycolysis / Gluconeogenesis biosynthesis pathway reconstructed based on the de novo assembly and annotation of the L. williamsii transcriptome. Figure S5. Starch and sucrose metabolic pathway in L.williamsii. Figure S7. Alignment of pyridoxal-dependent decarboxylase conserved domains in the deduced amino acid sequences of the UN13591 and UN15671 L. williamsii unigenes. Figure S8. (A) Alignment of the PPO1-KFDV C-terminal conserved domain of the UN14261 L. williamsii unigene and Glycine max PPO1 (XP_003522849.1). (B) Expression analysis of the L. williamsii unigene by RNA-seq. Figure S9. Real-time PCR validation of RNA-seq results. (PPTX 877 kb) [file 12864_2015_1821_MOESM2_ESM.pptx]

## Slide 1
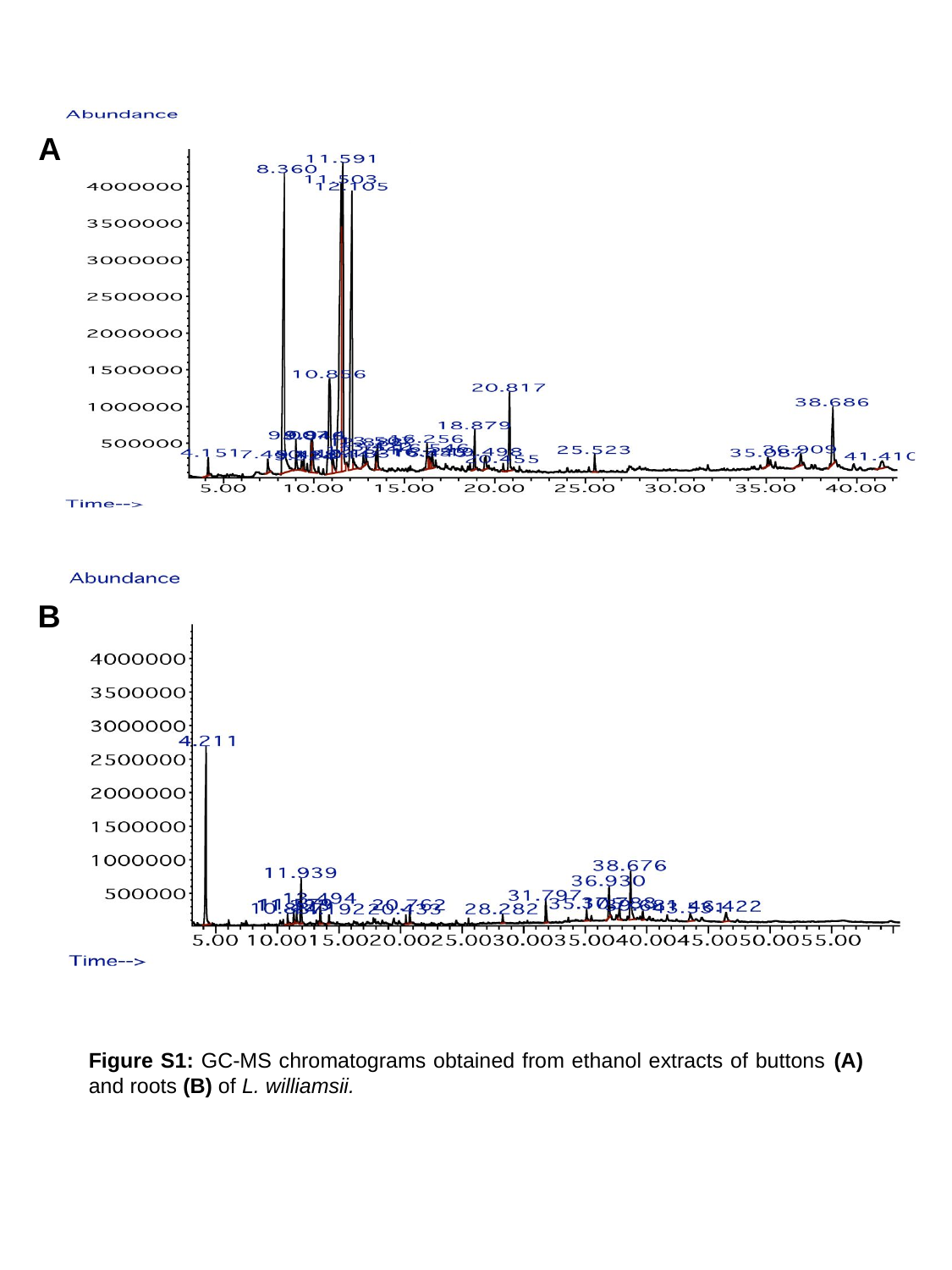

A
B
Figure S1: GC-MS chromatograms obtained from ethanol extracts of buttons (A) and roots (B) of L. williamsii.

## Slide 2
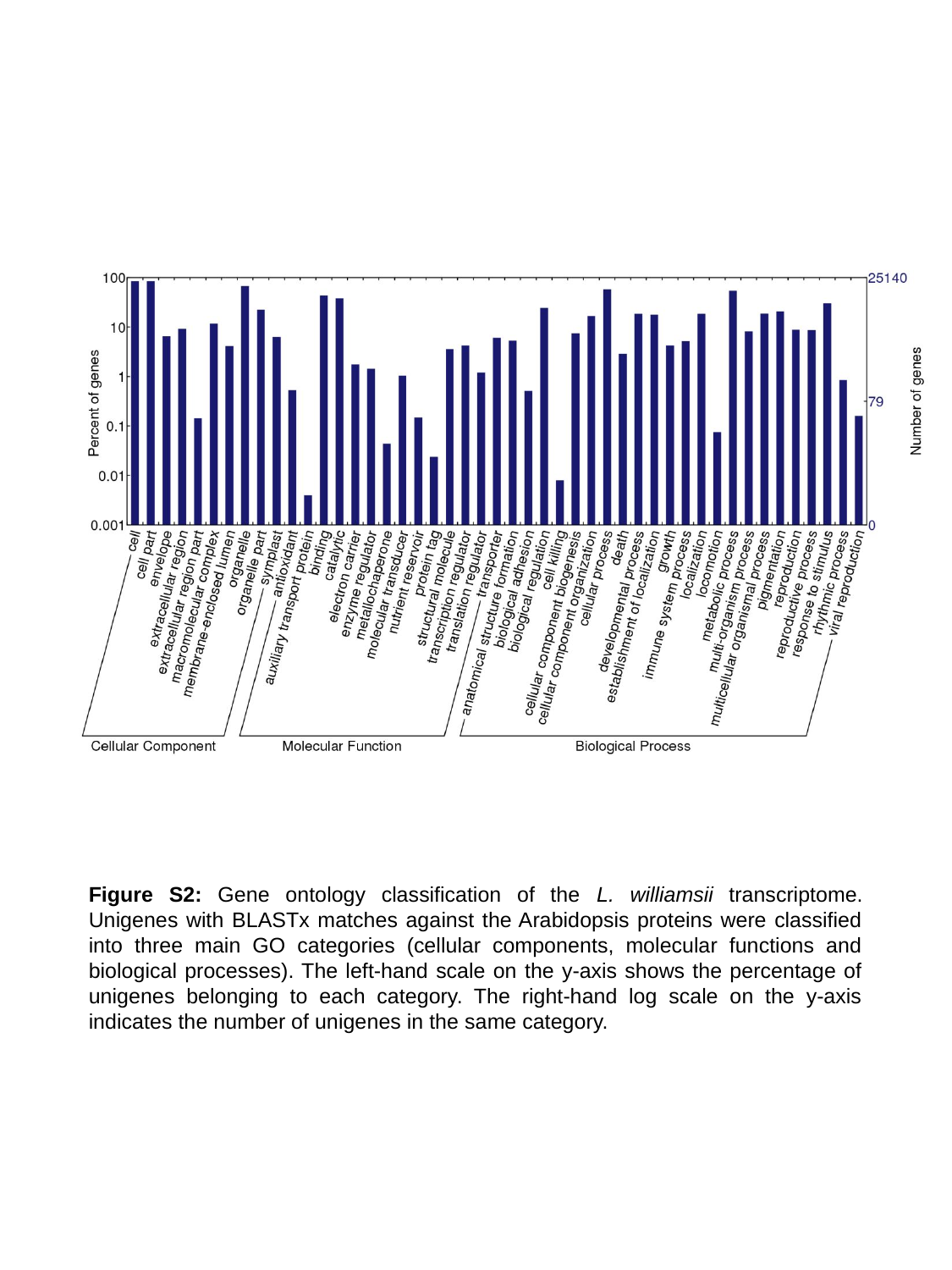

Figure S2: Gene ontology classification of the L. williamsii transcriptome. Unigenes with BLASTx matches against the Arabidopsis proteins were classified into three main GO categories (cellular components, molecular functions and biological processes). The left-hand scale on the y-axis shows the percentage of unigenes belonging to each category. The right-hand log scale on the y-axis indicates the number of unigenes in the same category.

## Slide 3
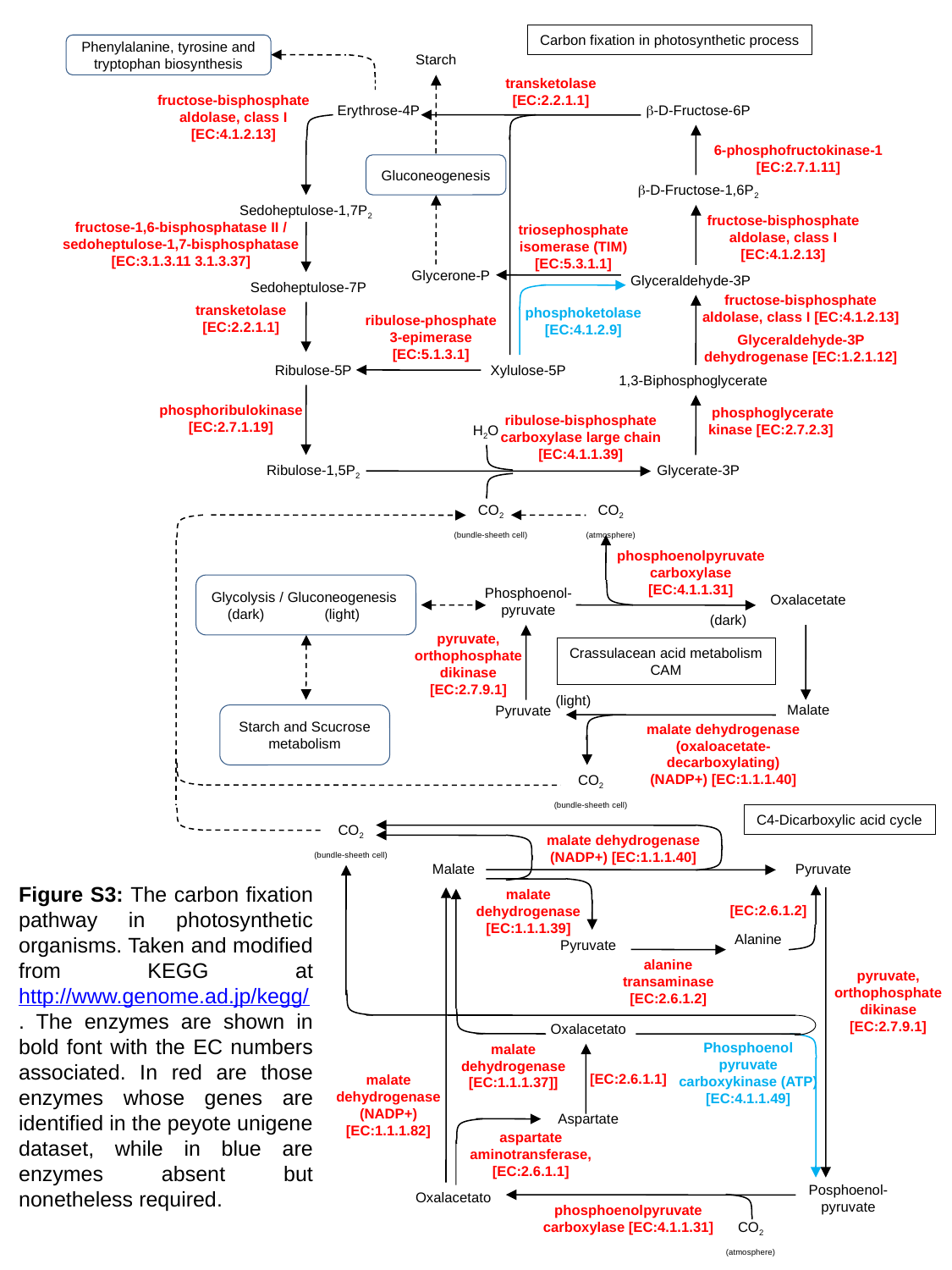

Carbon fixation in photosynthetic process
Phenylalanine, tyrosine and tryptophan biosynthesis
Starch
transketolase [EC:2.2.1.1]
fructose-bisphosphate aldolase, class I [EC:4.1.2.13]
Erythrose-4P
β-D-Fructose-6P
6-phosphofructokinase-1 [EC:2.7.1.11]
Gluconeogenesis
β-D-Fructose-1,6P2
Sedoheptulose-1,7P2
fructose-bisphosphate aldolase, class I [EC:4.1.2.13]
fructose-1,6-bisphosphatase II / sedoheptulose-1,7-bisphosphatase [EC:3.1.3.11 3.1.3.37]
triosephosphate isomerase (TIM) [EC:5.3.1.1]
Glycerone-P
Glyceraldehyde-3P
Sedoheptulose-7P
fructose-bisphosphate aldolase, class I [EC:4.1.2.13]
transketolase [EC:2.2.1.1]
phosphoketolase [EC:4.1.2.9]
ribulose-phosphate 3-epimerase [EC:5.1.3.1]
Glyceraldehyde-3P dehydrogenase [EC:1.2.1.12]
Ribulose-5P
Xylulose-5P
1,3-Biphosphoglycerate
phosphoribulokinase [EC:2.7.1.19]
 phosphoglycerate kinase [EC:2.7.2.3]
ribulose-bisphosphate carboxylase large chain [EC:4.1.1.39]
H2O
Ribulose-1,5P2
Glycerate-3P
CO2
(bundle-sheeth cell)
CO2
(atmosphere)
phosphoenolpyruvate carboxylase [EC:4.1.1.31]
Glycolysis / Gluconeogenesis
 (dark) (light)
Phosphoenol-pyruvate
Oxalacetate
(dark)
pyruvate, orthophosphate dikinase [EC:2.7.9.1]
Crassulacean acid metabolism
CAM
(light)
Malate
Pyruvate
Starch and Scucrose metabolism
malate dehydrogenase (oxaloacetate-decarboxylating) (NADP+) [EC:1.1.1.40]
CO2
(bundle-sheeth cell)
C4-Dicarboxylic acid cycle
CO2
(bundle-sheeth cell)
malate dehydrogenase (NADP+) [EC:1.1.1.40]
Pyruvate
Malate
malate dehydrogenase [EC:1.1.1.39]
[EC:2.6.1.2]
Alanine
Pyruvate
alanine transaminase [EC:2.6.1.2]
pyruvate, orthophosphate dikinase [EC:2.7.9.1]
Oxalacetato
Phosphoenol pyruvate carboxykinase (ATP) [EC:4.1.1.49]
malate dehydrogenase [EC:1.1.1.37]]
[EC:2.6.1.1]
malate dehydrogenase (NADP+) [EC:1.1.1.82]
Aspartate
aspartate aminotransferase, [EC:2.6.1.1]
Posphoenol-pyruvate
Oxalacetato
phosphoenolpyruvate carboxylase [EC:4.1.1.31]
CO2
(atmosphere)
Figure S3: The carbon fixation pathway in photosynthetic organisms. Taken and modified from KEGG at http://www.genome.ad.jp/kegg/. The enzymes are shown in bold font with the EC numbers associated. In red are those enzymes whose genes are identified in the peyote unigene dataset, while in blue are enzymes absent but nonetheless required.

## Slide 4
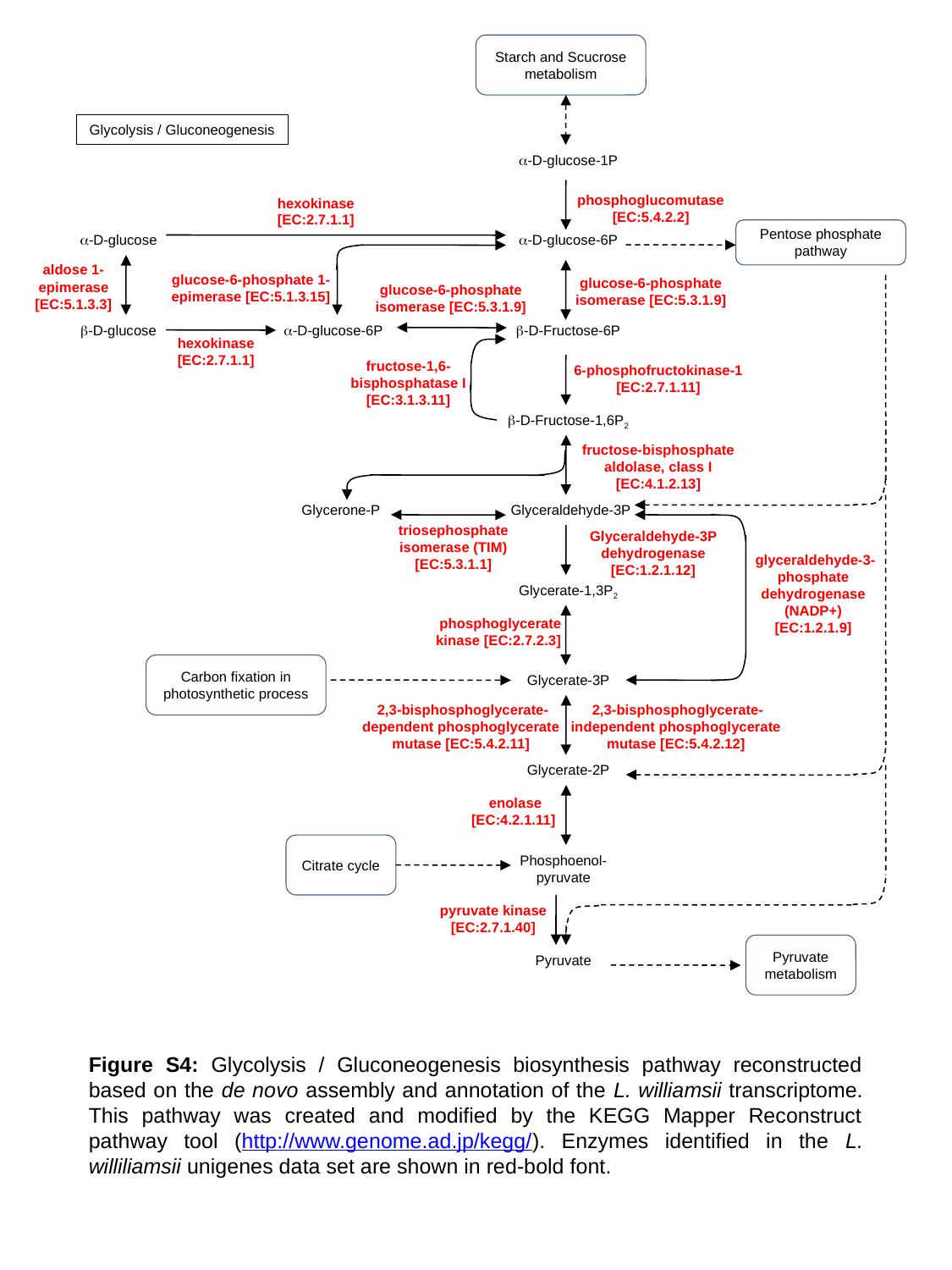

Starch and Scucrose metabolism
Glycolysis / Gluconeogenesis
α-D-glucose-1P
phosphoglucomutase [EC:5.4.2.2]
hexokinase [EC:2.7.1.1]
Pentose phosphate pathway
α-D-glucose-6P
α-D-glucose
aldose 1-epimerase [EC:5.1.3.3]
glucose-6-phosphate 1-epimerase [EC:5.1.3.15]
glucose-6-phosphate isomerase [EC:5.3.1.9]
glucose-6-phosphate isomerase [EC:5.3.1.9]
β-D-glucose
α-D-glucose-6P
β-D-Fructose-6P
hexokinase [EC:2.7.1.1]
fructose-1,6-bisphosphatase I [EC:3.1.3.11]
6-phosphofructokinase-1 [EC:2.7.1.11]
β-D-Fructose-1,6P2
fructose-bisphosphate aldolase, class I [EC:4.1.2.13]
Glycerone-P
Glyceraldehyde-3P
triosephosphate isomerase (TIM) [EC:5.3.1.1]
Glyceraldehyde-3P dehydrogenase [EC:1.2.1.12]
 glyceraldehyde-3-phosphate dehydrogenase (NADP+) [EC:1.2.1.9]
Glycerate-1,3P2
 phosphoglycerate kinase [EC:2.7.2.3]
Carbon fixation in photosynthetic process
Glycerate-3P
 2,3-bisphosphoglycerate-dependent phosphoglycerate mutase [EC:5.4.2.11]
 2,3-bisphosphoglycerate-independent phosphoglycerate mutase [EC:5.4.2.12]
Glycerate-2P
 enolase [EC:4.2.1.11]
Citrate cycle
Phosphoenol-pyruvate
pyruvate kinase [EC:2.7.1.40]
Pyruvate metabolism
Pyruvate
Figure S4: Glycolysis / Gluconeogenesis biosynthesis pathway reconstructed based on the de novo assembly and annotation of the L. williamsii transcriptome. This pathway was created and modified by the KEGG Mapper Reconstruct pathway tool (http://www.genome.ad.jp/kegg/). Enzymes identified in the L. williliamsii unigenes data set are shown in red-bold font.

## Slide 5
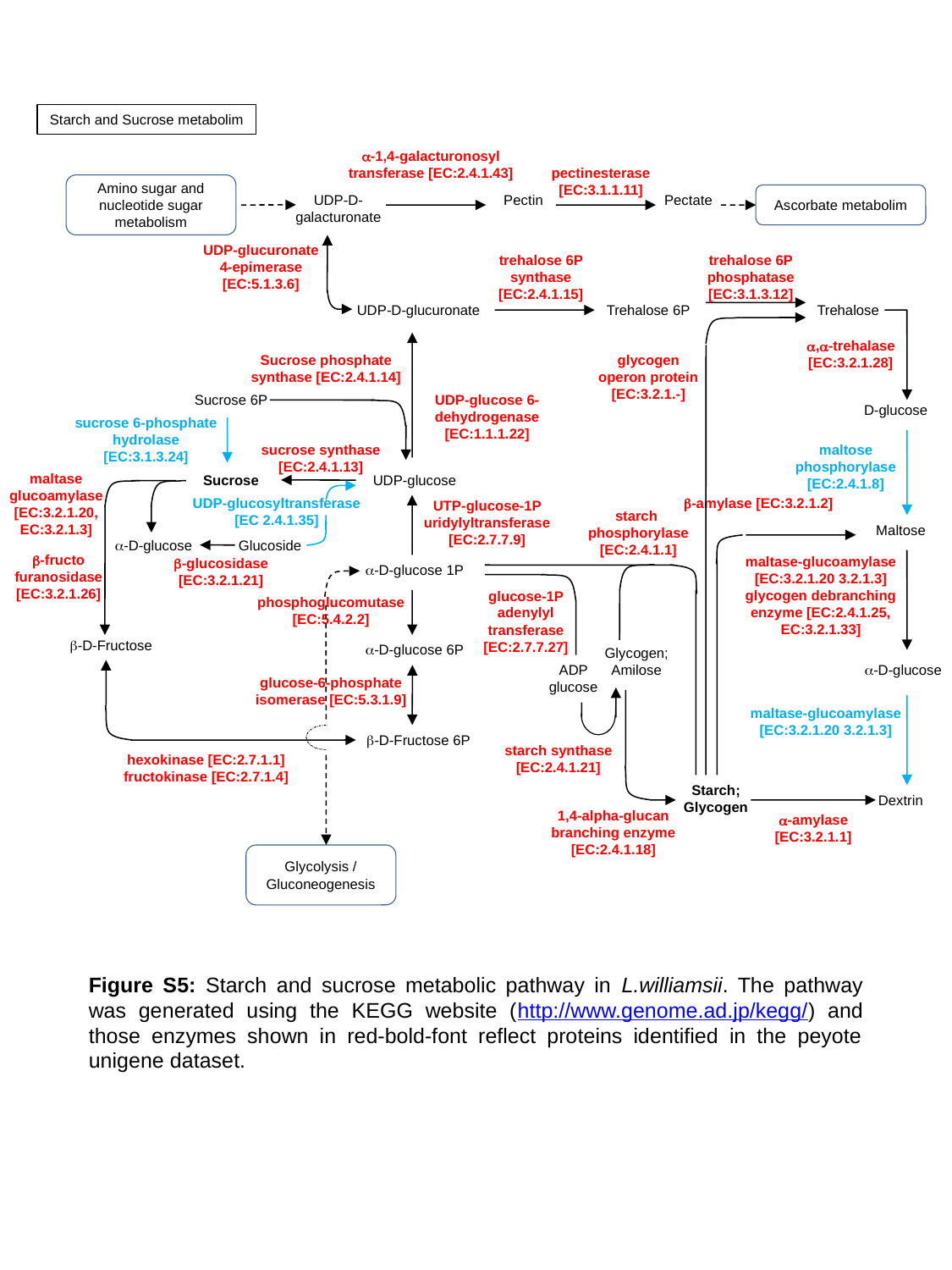

Starch and Sucrose metabolim
α-1,4-galacturonosyl transferase [EC:2.4.1.43]
pectinesterase [EC:3.1.1.11]
Amino sugar and nucleotide sugar metabolism
UDP-D-galacturonate
Pectin
Pectate
Ascorbate metabolim
UDP-glucuronate 4-epimerase
[EC:5.1.3.6]
trehalose 6P synthase [EC:2.4.1.15]
trehalose 6P phosphatase [EC:3.1.3.12]
UDP-D-glucuronate
Trehalose 6P
Trehalose
α,α-trehalase [EC:3.2.1.28]
Sucrose phosphate synthase [EC:2.4.1.14]
glycogen operon protein [EC:3.2.1.-]
Sucrose 6P
UDP-glucose 6-dehydrogenase [EC:1.1.1.22]
D-glucose
sucrose 6-phosphate hydrolase [EC:3.1.3.24]
sucrose synthase [EC:2.4.1.13]
maltose phosphorylase [EC:2.4.1.8]
maltase glucoamylase [EC:3.2.1.20, EC:3.2.1.3]
Sucrose
UDP-glucose
UDP-glucosyltransferase [EC 2.4.1.35]
β-amylase [EC:3.2.1.2]
UTP-glucose-1P uridylyltransferase [EC:2.7.7.9]
starch
phosphorylase [EC:2.4.1.1]
Maltose
α-D-glucose
Glucoside
β-fructo furanosidase [EC:3.2.1.26]
maltase-glucoamylase [EC:3.2.1.20 3.2.1.3]
glycogen debranching enzyme [EC:2.4.1.25, EC:3.2.1.33]
β-glucosidase [EC:3.2.1.21]
α-D-glucose 1P
glucose-1P adenylyl transferase [EC:2.7.7.27]
phosphoglucomutase [EC:5.4.2.2]
β-D-Fructose
α-D-glucose 6P
Glycogen; Amilose
ADP glucose
α-D-glucose
glucose-6-phosphate isomerase [EC:5.3.1.9]
maltase-glucoamylase [EC:3.2.1.20 3.2.1.3]
β-D-Fructose 6P
starch synthase [EC:2.4.1.21]
hexokinase [EC:2.7.1.1]
fructokinase [EC:2.7.1.4]
Starch; Glycogen
Dextrin
1,4-alpha-glucan branching enzyme [EC:2.4.1.18]
α-amylase [EC:3.2.1.1]
Glycolysis / Gluconeogenesis
Figure S5: Starch and sucrose metabolic pathway in L.williamsii. The pathway was generated using the KEGG website (http://www.genome.ad.jp/kegg/) and those enzymes shown in red-bold-font reflect proteins identified in the peyote unigene dataset.

## Slide 6
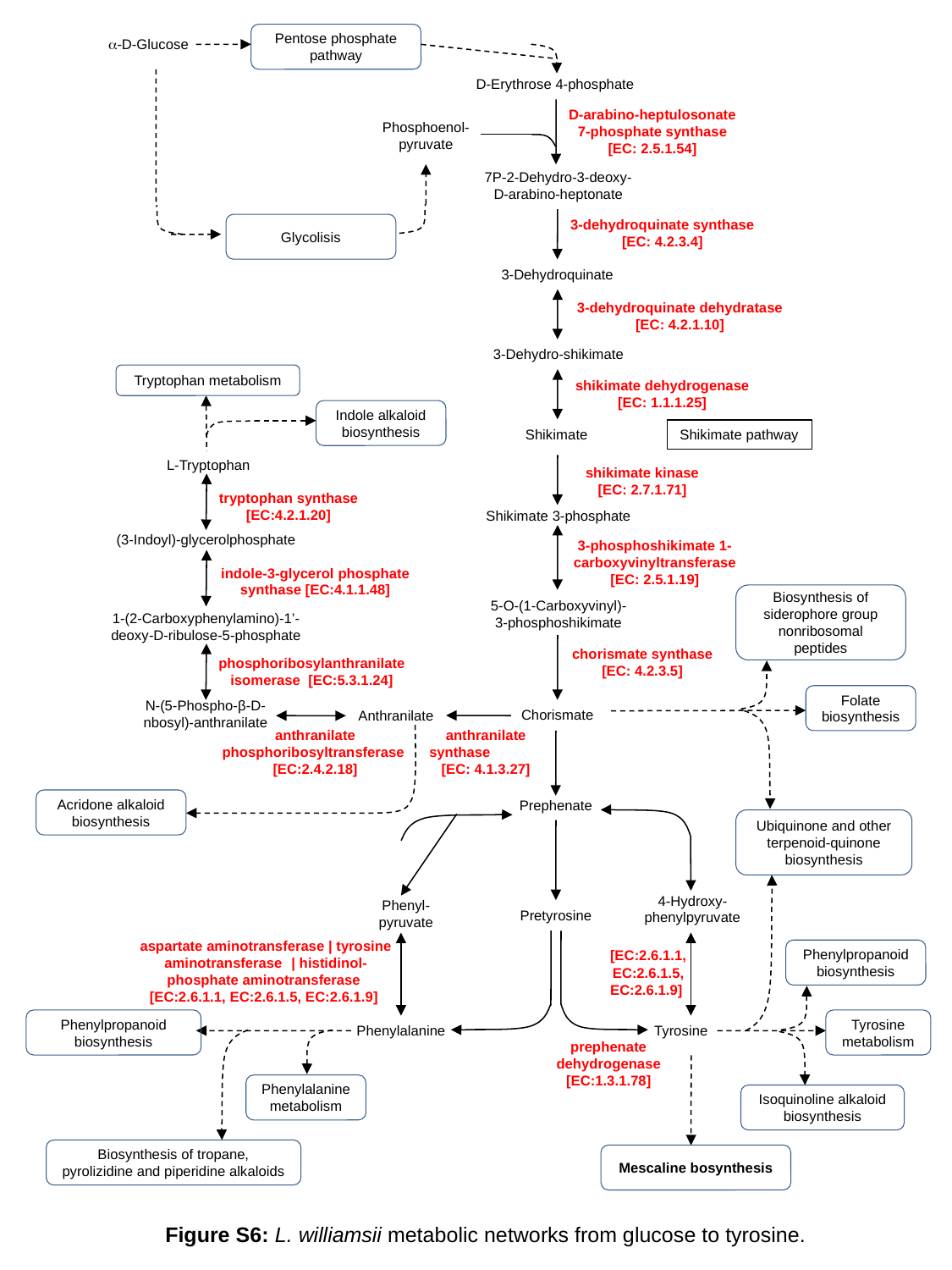

Pentose phosphate pathway
α-D-Glucose
D-Erythrose 4-phosphate
D-arabino-heptulosonate 7-phosphate synthase [EC: 2.5.1.54]
Phosphoenol-pyruvate
7P-2-Dehydro-3-deoxy-D-arabino-heptonate
3-dehydroquinate synthase [EC: 4.2.3.4]
Glycolisis
3-Dehydroquinate
3-dehydroquinate dehydratase [EC: 4.2.1.10]
3-Dehydro-shikimate
Tryptophan metabolism
shikimate dehydrogenase [EC: 1.1.1.25]
Indole alkaloid biosynthesis
Shikimate
Shikimate pathway
L-Tryptophan
shikimate kinase [EC: 2.7.1.71]
tryptophan synthase [EC:4.2.1.20]
Shikimate 3-phosphate
(3-Indoyl)-glycerolphosphate
3-phosphoshikimate 1-carboxyvinyltransferase [EC: 2.5.1.19]
indole-3-glycerol phosphate synthase [EC:4.1.1.48]
Biosynthesis of siderophore group nonribosomal peptides
5-O-(1-Carboxyvinyl)-3-phosphoshikimate
1-(2-Carboxyphenylamino)-1’-deoxy-D-ribulose-5-phosphate
chorismate synthase [EC: 4.2.3.5]
phosphoribosylanthranilate isomerase [EC:5.3.1.24]
Folate biosynthesis
N-(5-Phospho-β-D-nbosyl)-anthranilate
Chorismate
Anthranilate
anthranilate phosphoribosyltransferase [EC:2.4.2.18]
anthranilate synthase [EC: 4.1.3.27]
Acridone alkaloid biosynthesis
Prephenate
Ubiquinone and other terpenoid-quinone biosynthesis
4-Hydroxy-phenylpyruvate
Phenyl-pyruvate
Pretyrosine
aspartate aminotransferase | tyrosine aminotransferase 	| histidinol-phosphate aminotransferase [EC:2.6.1.1, EC:2.6.1.5, EC:2.6.1.9]
Phenylpropanoid biosynthesis
[EC:2.6.1.1, EC:2.6.1.5, EC:2.6.1.9]
Phenylpropanoid biosynthesis
Tyrosine metabolism
Phenylalanine
Tyrosine
prephenate dehydrogenase [EC:1.3.1.78]
Phenylalanine metabolism
Isoquinoline alkaloid biosynthesis
Biosynthesis of tropane, pyrolizidine and piperidine alkaloids
Mescaline bosynthesis
Figure S6: L. williamsii metabolic networks from glucose to tyrosine.

## Slide 7
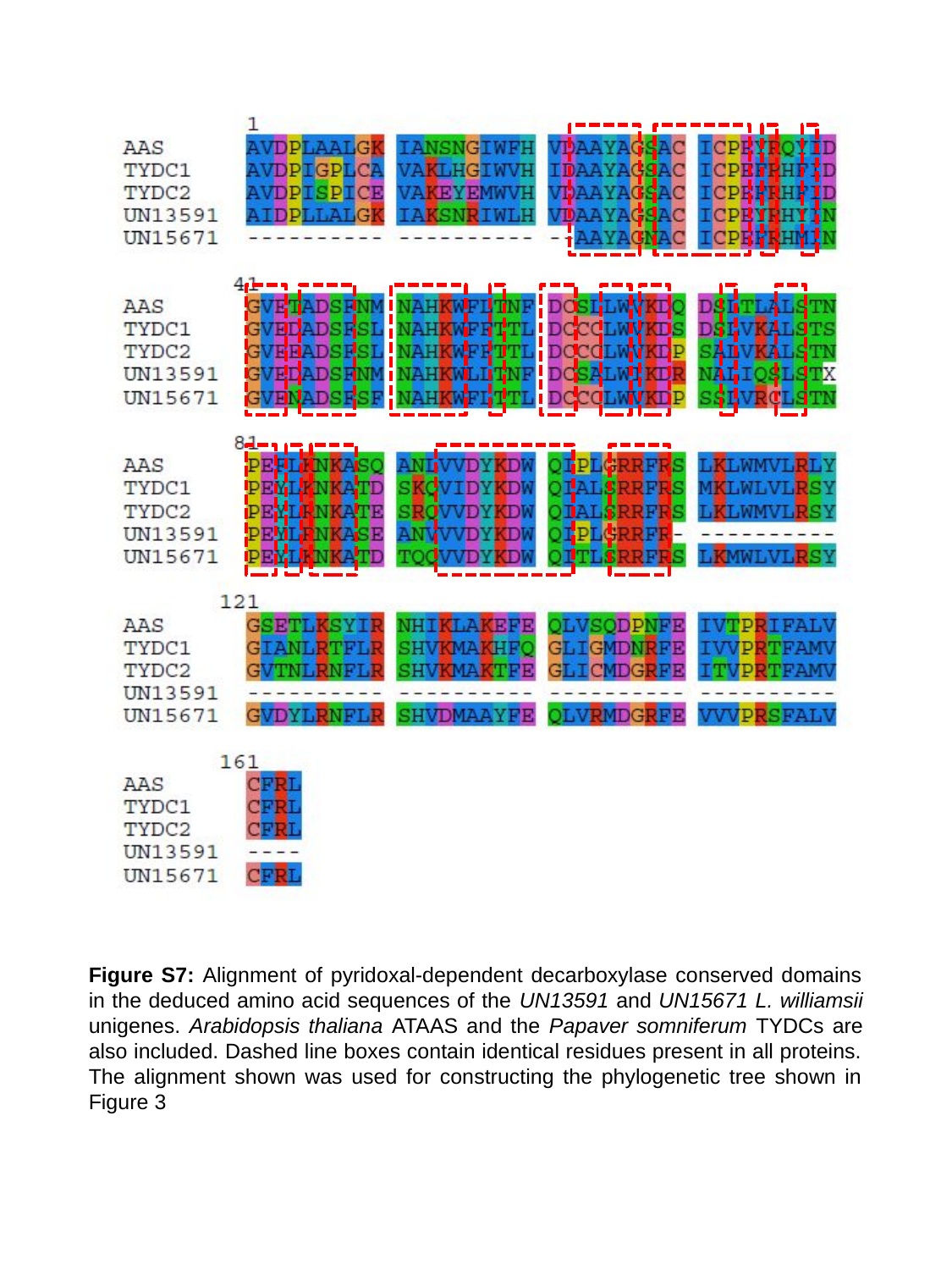

Figure S7: Alignment of pyridoxal-dependent decarboxylase conserved domains in the deduced amino acid sequences of the UN13591 and UN15671 L. williamsii unigenes. Arabidopsis thaliana ATAAS and the Papaver somniferum TYDCs are also included. Dashed line boxes contain identical residues present in all proteins. The alignment shown was used for constructing the phylogenetic tree shown in Figure 3

## Slide 8
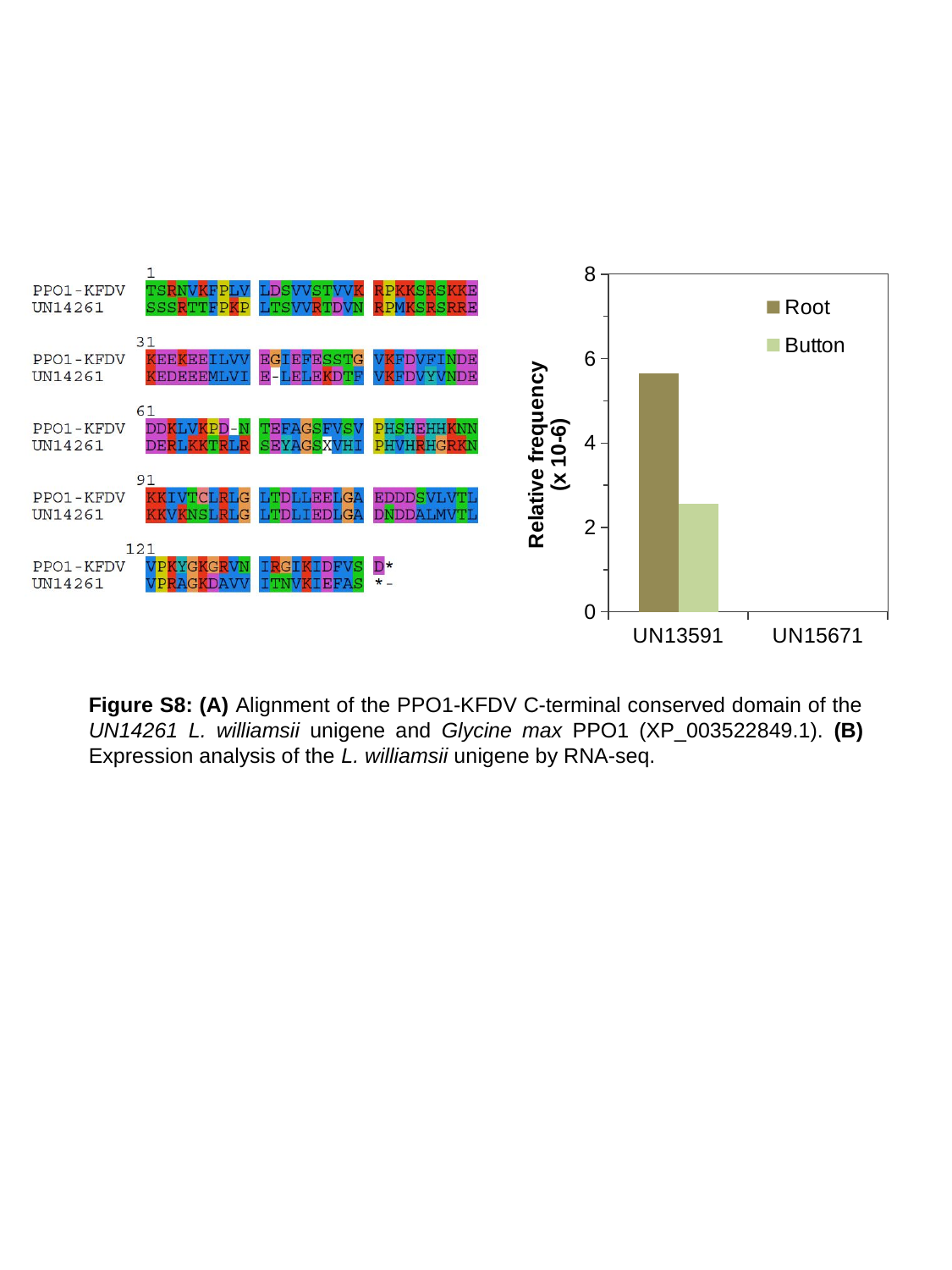

[unsupported chart]
Figure S8: (A) Alignment of the PPO1-KFDV C-terminal conserved domain of the UN14261 L. williamsii unigene and Glycine max PPO1 (XP_003522849.1). (B) Expression analysis of the L. williamsii unigene by RNA-seq.

## Slide 9
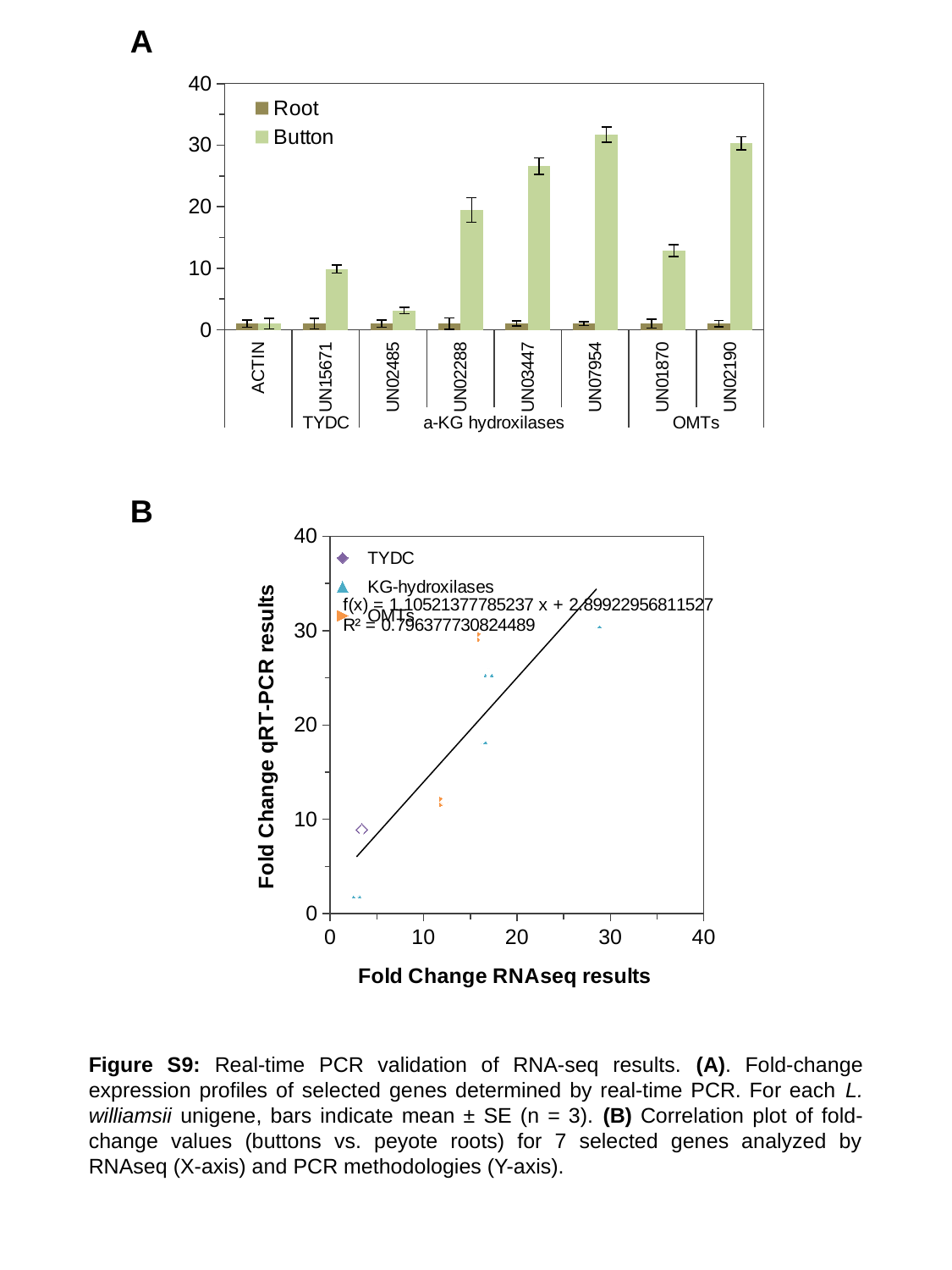

A
### Chart
| Category | Root | Button |
|---|---|---|
| ACTIN | 1.0 | 1.0 |
| UN15671 | 1.0 | 9.887981886584305 |
| UN02485 | 1.0 | 3.109496836527234 |
| UN02288 | 1.0 | 19.49083612626759 |
| UN03447 | 1.0 | 26.598645899233507 |
| UN07954 | 1.0 | 31.727580818504286 |
| UN01870 | 1.0 | 12.844347358020086 |
| UN02190 | 1.0 | 30.324399726279374 |B
### Chart
| Category | | | | |
|---|---|---|---|---|Figure S9: Real-time PCR validation of RNA-seq results. (A). Fold-change expression profiles of selected genes determined by real-time PCR. For each L. williamsii unigene, bars indicate mean ± SE (n = 3). (B) Correlation plot of fold-change values (buttons vs. peyote roots) for 7 selected genes analyzed by RNAseq (X-axis) and PCR methodologies (Y-axis).
